# Modulating Ion‐Dipole and Dipole–Dipole Interactions for Stable Wide‐Temperature‐Range Lithium–Sulfur Batteries Enabled by Quantum‐Dot Catalysts

**DOI:** 10.1002/anie.202512168

**Published:** 2025-08-05

**Authors:** Yongqian He, Duanfeng Xiong, Manfang Chen, Wanqi Zhang, Sisi Liu, Yongjie Ye, Mengqing Wang, Ying Chen, Qin Tang, Xuewen Peng, Caixiang Wang, Hongyang Zhan, Hong Liu, Min Liu, Jincang Su, Hongbo Shu, Jian Wang, Xianyou Wang

**Affiliations:** ^1^ National Base for International Science & Technology Cooperation of New Energy Equipment, Energy Storage Materials and Devices, National Local Joint Engineering Laboratory for Key Materials of New Energy Storage Battery, Hunan Province Key Laboratory of Electrochemical Energy Storage & Conversion, School of Chemistry Xiangtan University Xiangtan Hunan 411105 China; ^2^ College of New Energy Ningbo University of Technology Ningbo Zhejiang 315336 China; ^3^ School of Materials Science and Engineering Xiangtan University Xiangtan 411105 China; ^4^ Helmholtz Institute Ulm (HIU) Ulm D89081 Germany; ^5^ Karlsruhe Institute of Technology (KIT) Karlsruhe D‐76021 Germany

**Keywords:** Broad temperature range, Capacity decay, Dipole–dipole interaction, Ion‐dipolar effect, Lithium–sulfur batteries

## Abstract

The incomplete conversion of sulfur species, particularly the pivotal intermediate solid Li_2_S_2_ during redox processes, poses a significant limitation on the cyclability of lithium–sulfur batteries (LSBs). Herein, a synergistic modulation strategy of ion‐/dipole–dipole interactions that tailors the solvation sheath configuration and activates the electrochemical reactivity of Li_2_S_2_ is initially proposed for accelerating kinetics. As a proof of concept, the molybdenum nitride quantum dots located on nitrogen‐doped carbon (MoNQDs/NC) were designed. Advanced in situ/ex situ characterizations combined with theoretical calculations reveal that MoNQDs/NC effectively weaken the ion‐dipole interactions within Li(solvent)*_x_* ^+^ species, thereby facilitating the desolvation process. Furthermore, the robust dipole–dipole interactions between polar domains of MoNQDs and Li_2_S_2_ are realized to generate localized tensile strain fields to destabilize the S─S/Li─S bonds network. Consequently, the optimal cells maintain a high areal capacity (>5.0 mAh cm^−2^) after 50 cycles at high sulfur loading (4.4–9.1 mg cm^−2^) over a wide temperature range (0–60 °C). Furthermore, the pouch cell with a sulfur loading of 1.5 g retained a capacity of 1.79 Ah after 15 cycles, highlighting the potential of this ion‐dipole modulation strategy for commercializing LSBs.

## Graphical abstract

The electrochemical inertness of Li_2_S_2_ leads to irreversible sulfur loss. MoNQDs/NC reconfigure the solvation shell structure, thereby weakening the ion‐dipole effect within Li(solvent)*_x_* ^+^ and enabling a fast desolvation process. Furthermore, localized dipole fields from their ordered Mo–N atoms enhance dipole–dipole interactions with Li_2_S_2_. This induces tensile strain, destabilizing Li_2_S_2_ and promoting its decomposition. Via this dual mechanism, MoNQDs/NC enable high‐sulfur‐loading LSBs to achieve low capacity decay over a wide temperature range.

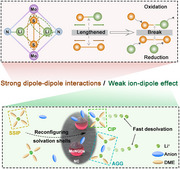

## Introduction

Lithium–sulfur batteries (LSBs) occupy a pivotal position among next‐generation secondary battery technologies due to their high theoretical specific capacity (1675 mAh g^−1^), the abundance of sulfur, and minimal environmental impact.^[^ ^1^ ^]^ However, suboptimal practical capacity reversibility has hindered the commercialization progress of LSBs. Traditionally, this has been attributed to the shuttle effect of soluble lithium polysulfides (LiPSs) and the extremely low conductivity of the discharge product Li_2_S, leading to inefficient reutilization of sulfur species.^[^ ^2^, ^3^ ^]^ Consequently, extensive research has focused on prohibiting the shuttle effect or lowering the nucleation/decomposition barriers of Li_2_S by introducing various electrocatalysts, such as conductive carbon materials, polar non‐metal heteroatoms, and transition metal single atoms.^[^ ^4^, ^5^, ^6^, ^7^ ^]^ These efforts have somewhat improved LSBs performance. However, the persistent capacity fading during cycling, particularly the performance deterioration under extended temperature windows, remains a critical bottleneck hindering the development of LSBs.^[^ ^8^ ^]^ Most importantly, this indicates the existence of fundamental regulatory mechanisms at the electrode–electrolyte interface and the kinetics of sulfur conversion that have not yet been fully elucidated.

**Figure 1:**
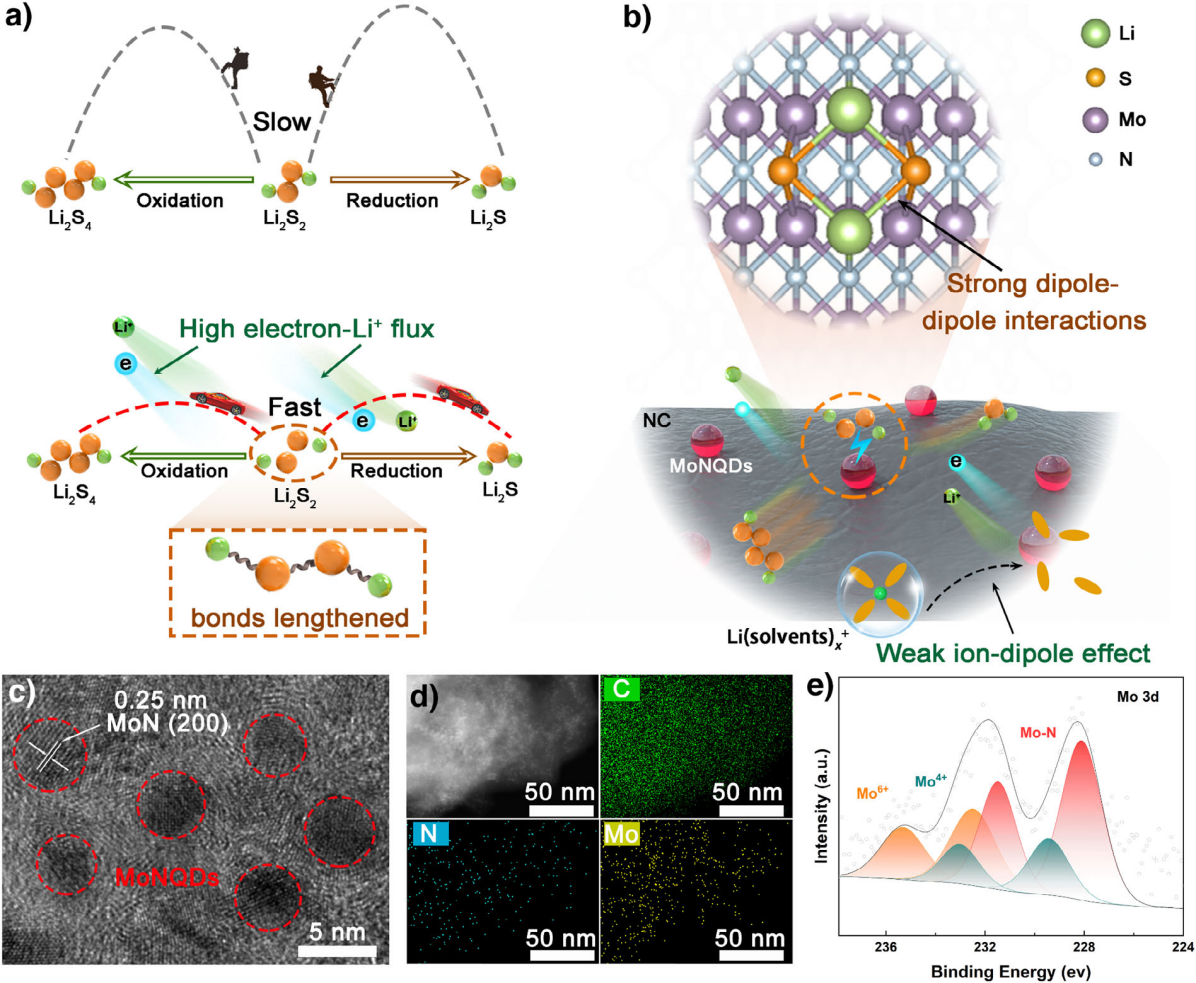
a) Conversion mechanism diagram of Li_2_S_2_. b) Schematic illustration of the modification mechanism of MoNQDs/NC in LSBs. c) HRTEM and d) EDX images of MoNQDs/NC. e) XPS spectra of Mo 3d of MoNQDs/NC.

Substantial experimental evidence has established the inherently restricted solubility of LiPSs in conventional ether‐based electrolytes, a fundamental constraint that persists across operational battery configurations.^[^ ^9^, ^10^, ^11^ ^]^ Moreover, advanced catalytic systems have demonstrated efficient conversions of LiPSs into solid‐phase Li_2_S_2_/Li_2_S during sulfur reduction reactions (SRR), thereby establishing an intrinsic self‐limiting mechanism against LiPSs migration.^[^ ^12^, ^13^ ^]^ Recent mechanistic investigations further reveal that while Li_2_S exhibits intrinsically sluggish electrochemical oxidation kinetics, its chemical interaction with dissolved LiPSs during sulfur oxidation reactions (SOR) generates reactive intermediates that bypass traditional electrochemical pathways. This chemical‐electrochemical coupling effect ensures continuous Li_2_S utilization without requiring direct electrochemical oxidation.^[^ ^14^, ^15^ ^]^ Guided by these observations, advanced operando characterizations have identified Li_2_S_2_ as a persistent metastable phase throughout the sulfur redox process, with incomplete electrochemical decomposition observed during charge processes.^[^ ^16^ ^]^ The electrochemical inertness of Li_2_S_2_ leads to dual detrimental effects: active sulfur loss and catalyst deactivation due to surface passivation by inactive sulfur species. Therefore, while ensuring high catalytic activity of electrocatalysts for both SRR and SOR, their impacts on Li_2_S_2_ decomposition kinetics should also be considered, which is crucial for enhancing the capacity reversibility of LSBs. The electrochemical decomposition of Li_2_S_2_ is theoretically predicted to encounter dual kinetic limitations: (1) the endergonic cleavage of S─S/Li─S covalent bonds requiring substantial activation energy and (2) the energetically demanding Li^+^ desolvation process during phase transfer.^[^ ^17^, ^18^, ^19^, ^20^ ^]^ An effective interaction for this process must simultaneously destabilize Li_2_S_2_ through strong interfacial interactions and provide lithiophilic sites with minimized energy barriers for Li^+^ desolvation and diffusion to facilitate charge transfer (Figure 1a). However, establishing clear structure‐activity relationships for Li–S systems remains critically underexplored, especially when operating under extreme electrochemical conditions.

Herein, a synergistic modulation strategy of ion/dipole–dipole interactions that tailors the solvation sheath configuration and activates the electrochemical reactivity of Li_2_S_2_ is initially proposed for accelerating kinetics. Generally, considering the unique advantages of quantum dots (QDs) in sulfur electrocatalyst, which include atomic‐scale size and highly active edge catalytic sites,^[^ ^21^, ^22^, ^23^ ^]^ the 2D nitrogen‐doped carbon substrate embedded with molybdenum nitride QDs (MoNQDs/NC) has been strategically synthesized via a high‐temperature nitridation process. Integrated with density functional theory (DFT) calculations, COMSOL Multiphysics simulations, and advanced in situ/ex situ characterizations, the MoNQDs/NC modulates the Li(solvents)*_x_* ^+^ structure at the electrode–electrolyte interface. Simultaneously, the periodic Mo–N atomic arrangement in MoNQDs generates localized dipole fields that impose directional tensile strain on Li_2_S_2_ molecules through intensified dipole–dipole interactions, thereby destabilizing their molecular configuration and reducing the energy barrier for Li─S/S─S bonds cleavage, significantly enhancing sulfur redox reversibility (Figure 1b). Consequently, the cell with MoNQDs/NC‐functionalized separators achieves an initial areal capacity of 8.3 mAh cm^−2^ at a high sulfur loading of 9.1 mg cm^−2^ with a capacity retention rate of 99.4%. Even under the harsh conditions of 0 and 60 °C, the high‐loading cell maintains an excellent average capacity (>5.0 mAh cm^−2^) at 0.2 C. Moreover, the lean‐electrolyte pouch cell (E/S ratio ≈ 4.0 µL mg^−1^) with a high sulfur loading of 1.5 g exhibited a remarkable discharge capacity of 1.87 Ah, establishing an interfacial ion/dipole co‐regulation paradigm for practical LSBs.

## Results and Discussion

First, Figure S1 illustrates the preparation process of MoNQDs/NC. Briefly, through a hydrothermal reaction, molybdenum precursors are uniformly dispersed on the surface of melamine. In a tubular furnace, the molybdenum precursors are then nitrided using NH_3_ generated from the pyrolysis of hexamethylenetetramine, yielding the target material MoNQDs/NC. The morphology and structure of the material are characterized by scanning electron microscopy (SEM) and transmission electron microscopy (TEM). SEM results indicate that the MoNQDs do not disrupt the structure of NC, and the material exhibits a two‐dimensional morphology (Figure S2a,b). TEM and high‐resolution TEM (HRTEM) images confirm the uniform distribution of MoNQDs on NC, while statistical analysis reveals that 96% of these QDs exhibit sizes below 5 nm with an average diameter of 3.6 nm, demonstrating the successful synthesis of QDs of the target size (Figure S2c,d). Figure 1c shows that the lattice fringe spacing is 0.25 nm, which corresponds to the (200) plane of MoN.^[^ ^24^ ^]^ Further analysis of the crystal structure of the sample is conducted through X‐ray diffraction (XRD). As shown in Figure S3a, the XRD pattern of MoNQDs/NC exhibits weak characteristic peaks at 31.9°, 36.2°, and 49.0°, attributed to the (002), (200), and (202) planes of MoN, respectively. These peaks are likely shielded by the peaks of the carbon material. The presence of these characteristic planes is confirmed by the corresponding diffraction rings observed in selected area electron diffraction (SAED) (Figure S3b). Additionally, energy‐dispersive X‐ray (EDX) spectrum demonstrates the uniform distribution of N, C, and Mo elements in MoNQDs/NC (Figure 1d). The chemical states of the elements are further characterized by X‐ray photoelectron spectroscopy (XPS). The C 1s spectrum exhibits five peaks at 284.2, 284.8, 285.4, 286.4, and 288.8 eV, corresponding to Mo─C, C─C/C═C, C─N, C─O, and O─C─O, respectively (Figure S4a).^[^ ^25^ ^]^ The N 1s spectrum can be deconvoluted into four peaks, assigned to Mo─N bonds (398.1 eV), pyridinic N (399.5 eV), pyrrolic N (400.8 eV), and graphitic N (402.5 eV) (Figure S4b).^[^ ^24^, ^26^ ^]^ In the nitrogen‐doped configuration, the relative contents of pyridinic N, pyrrolic N, and graphitic N are 17%, 68%, and 15%, respectively. Considering the predominant contribution of pyrrolic N, it serves as the primary structural motif for modeling the NC support in subsequent DFT calculations. As shown in Figure 1e, the Mo 3d spectrum can be deconvoluted into Mo^3+^, Mo^4+^, and Mo^6+^ species. The peaks at 228.1 and 231.5 eV are attributed to Mo^3+^ in MoN, while the peaks assigned to Mo^4+^ and Mo^6+^ are due to inevitable surface oxidation.^[^ ^21^, ^24^ ^]^ These results confirm the successful preparation of MoNQDs/NC. Furthermore, to explore the potential of functional materials as electrocatalysts, N_2_ isothermal adsorption/desorption tests are employed to analyze the pore distribution and surface area of the materials. The results indicate that, compared to NC, the MoNQDs/NC sample exhibits a relatively large specific surface area of 54.53 m^2^ g^−1^ and a high pore volume of 0.248 cm^3^ g^−1^. Notably, the prepared samples all exhibit abundant micro‐ and mesoporous structures, which enable efficient entrapment and stabilization of active species (Figure S5a,b). The electronic conductivity of the material is evaluated using a four‐probe conductivity meter. As shown in Figure S5c,d, the conductivity of MoNQDs/NC exceeds that of NC by three orders of magnitude, suggesting that MoNQDs optimize the conductive network of the NC carrier, which will facilitate faster electron transfer for sulfur electrochemical reactions.

The adsorption capacity of functional materials toward LiPSs has a significant impact on the subsequent conversion processes. Therefore, visual adsorption experiments were conducted to explore this aspect. As shown in the ultraviolet–visible (UV–vis) spectrum and its inset in Figure S6a, MoNQDs/NC exhibited the highest adsorption capability. XPS analysis can further elucidate the adsorption mechanism of MoNQDs/NC. The shift of peak positions to higher energy levels in the Mo 3d spectrum confirmed that during the chemical adsorption process with Li_2_S_6_, Mo atoms tend to transfer electrons to S atoms (Figure S6b). Additionally, the signal peak of the N─Li bond further indicated the presence of chemical interactions between N atoms and Li atoms (Figure S6c).^[^ ^24^ ^]^ In Figure S6d, the XPS spectrum of S 2p could be deconvolved into sulfur‐metal bond, terminal sulfur (S_T_^−1^), bridged sulfur (S_B_^0^), thiosulfate, and polythionate species.^[^ ^27^ ^]^ These results all validate the excellent anchoring effect of MoNQDs/NC on LiPSs, which is beneficial for inhibiting the shuttle effect and enhancing subsequent sulfur conversion kinetics. As shown in Figure S7, the exchange current density of MoNQDs/NC‐based symmetric cell involving Li_2_S_6_ solution was greater than that of NC‐based symmetric cell, implying that MoNQDs/NC can effectively promote the conversion of LiPSs due to its excellent adsorption ability.

**Figure 2:**
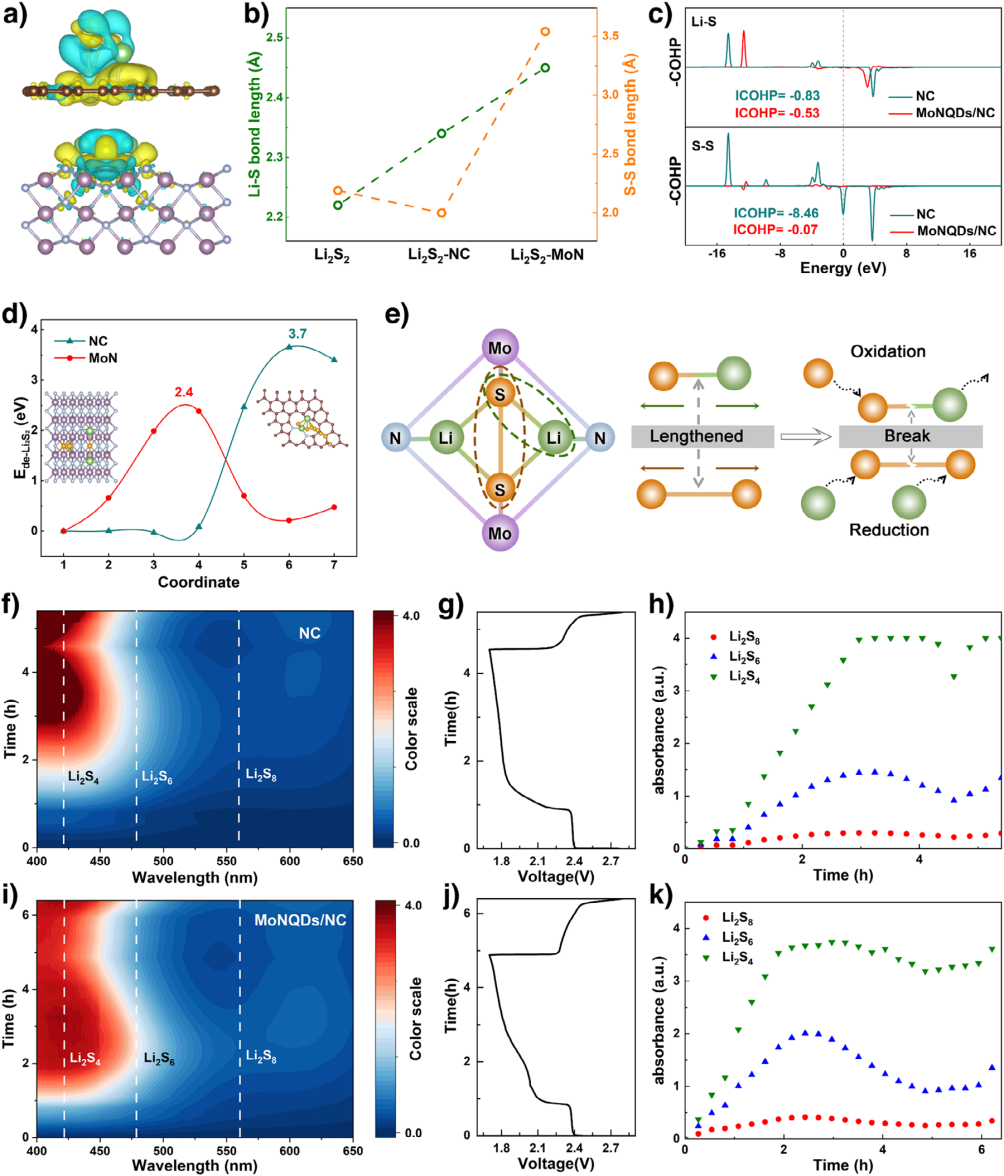
a) Adsorption models of Li_2_S_2_ on NC and MoN substrate. b) Comparison of bond lengths of Li─S/S─S bonds of Li_2_S_2_ adsorbed. c) COHPs of Li─S/S─S bonds of Li_2_S_2_ adsorbed. d) Energy profiles of the decomposition of Li_2_S_2_. e) Schematic representation of the change in the chemical structure of Li_2_S_2_ under tensile strain of MoN. f) Contour maps of in situ UV–vis spectra, g) the corresponding GCD curve and h) absorbance data of the dissolved LiPSs for the cell with NC. i) Contour maps of in situ UV–vis spectra, j) the corresponding GCD curve and k) absorbance data of the dissolved LiPSs for the cell with MoNQDs/NC.

DFT calculations were then used to simulate the adsorption configuration of Li_2_S_2_ as a key sulfur species on the catalyst (Figure 2a). As demonstrated in Figures 2b and S8, the periodic Mo–N atomic arrangement within MoN generates localized dipole fields, in stark contrast to the pristine Li_2_S_2_ species and those adsorbed on the NC substrate. These dipole fields exert directional tensile strain on the Li_2_S_2_ molecules through intensified dipole–dipole interactions, ultimately leading to the elongation of the Li─S/S─S bonds within the Li_2_S_2_ structure. Furthermore, the higher adsorption energy of MoN toward Li_2_S_2_ compared to NC also confirms the strong dipole interaction (−6.1 eV versus −3.9 eV) (Figure S9). Further analysis of the Crystal Orbital Hamilton Population (COHP) for the Li─S/S─S bonds in Li_2_S_2_ under the adsorption of two catalysts is shown in Figure 2c. The positive region of the COHP curve corresponds to bonding states, while the negative region corresponds to antibonding states. The bonding strength can be quantitatively evaluated by the integrated COHP (ICOHP) below the Fermi level. The ICOHP values for Li─S (S─S) bonds in the NC and MoN systems are determined to be −0.83 (−8.46) and −0.53 (−0.07), respectively. These results suggest that the bonding interactions of Li─S and S─S bonds are significantly weakened under the strong dipole field‐induced stretching effect of MoN. The decomposition behavior of Li_2_S_2_ was then simulated, and the results showed that the energy barrier of the decomposition process of Li_2_S_2_ on MoN was lower compared to that on NC (2.4 eV versus 3.7 eV), thus confirming the optimization of the decomposition process of Li_2_S_2_ by MoN (Figure 2d). Therefore, under the tensile strain induced by MoN, both Li─S and S─S bonds in the Li_2_S_2_ molecule are stretched, and its structural stability is greatly reduced, which is conducive to the simultaneous enhancement of the kinetics of Li_2_S_2_ during the oxidation and reduction processes (Figure 2e).

To further investigate the mechanism of action of MoNQDs/NC on sulfur redox processes, in situ UV–vis spectroscopy was conducted to measure the real‐time concentration of soluble LiPSs (Figure 2f,i). The corresponding time‐potential profiles for different materials were compared in Figure 2g,j. It is evident that batteries with MoNQDs/NC exhibit longer discharge and charge times compared to those with NC, indicating higher utilization efficiency of sulfur species. Subsequently, the characteristic absorbances at 420 nm (for Li_2_S_4_), 480 nm (for Li_2_S_6_), and 560 nm (for Li_2_S_8_) were used as benchmarks to illustrate the state of polysulfide dissolution after dissolution (Figure 2h,k).^[^ ^25^ ^]^ It can be observed that the concentration change of LiPSs in MoNQDs/NC battery is more rapid, representing enhanced sulfur conversion kinetics. It is noteworthy that in Figure 2k, the concentration of Li_2_S_4_ starts to rise after an initial decrease in the discharge time around 4 h, which stems from the unstable disproportionation decomposition of Li_2_S_2_.^[^ ^16^ ^]^ The relevant reaction process is shown below:^[^ ^28^, ^29^ ^]^

(1) $$\;\;{\text{3S}}_{2}^{2-}\;\;↔\;\;2S^{2-}\;\;+\;\;S_{4}^{2-}$$

(2) $$\;\;{\text{2S}}_{4}^{2-}\;\;↔\;\;S_{2}^{2-}\;\;+\;\;S_{6}^{2-}$$

These results further confirm the rationality and superiority of the strong dipole fields of MoNQDs in achieving the efficient utilization of Li_2_S_2_.

**Figure 3:**
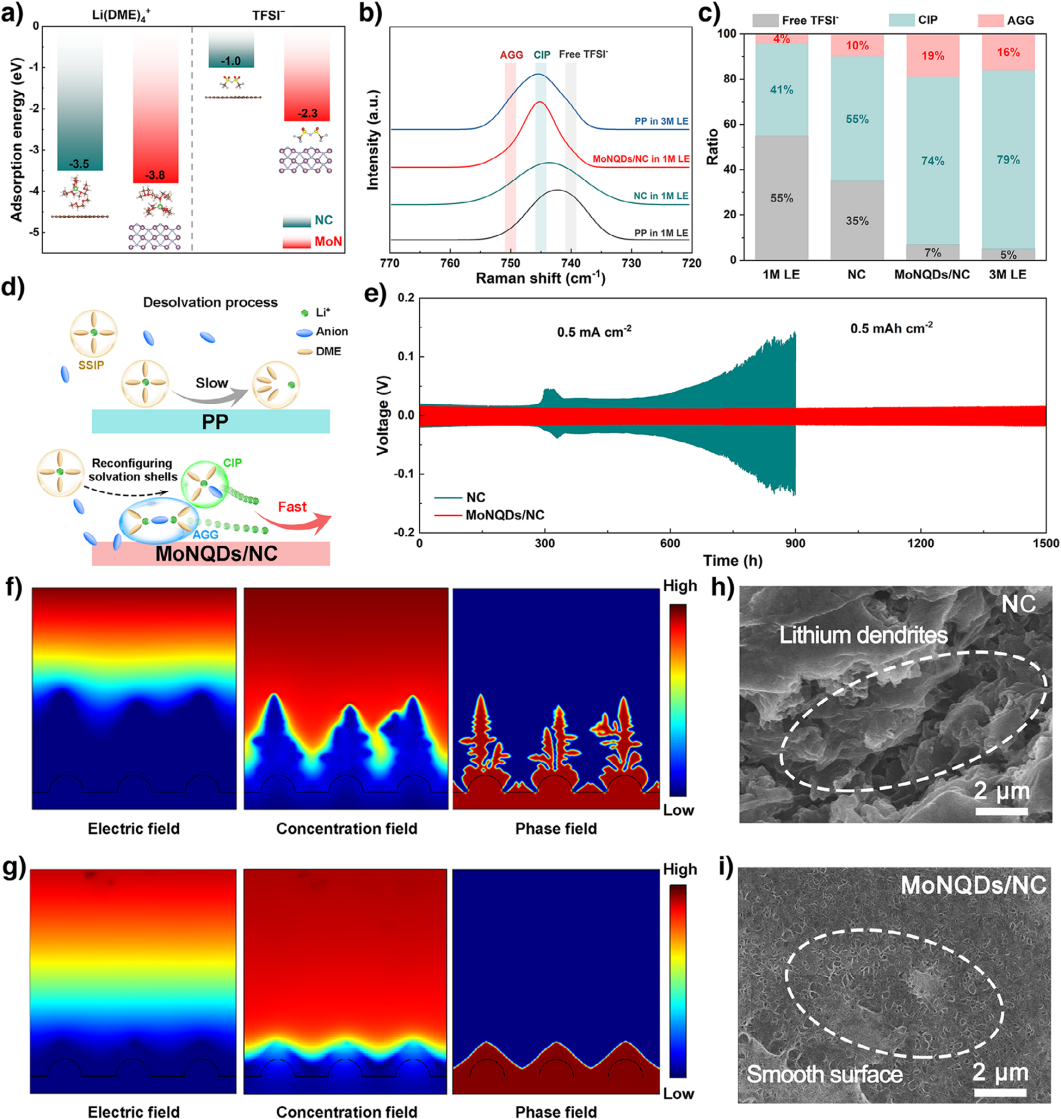
a) Adsorption energies of Li(DME)_4_^+^ and TFSI^−^ on different materials. b) Raman spectra of the various electrolyte with/without MoNQDs/NC. c) The ratio summary of free TFSI^−^, CIP, and AGG in different systems. d) Schematic comparison of desolvation processes for Li(solvent)*_x_* ^+^. e) Cycling performance of Li||Li symmetric cells. COMSOL results with f) NC and g) MoNQDs/NC separators. SEM images of Li anode after cycling of the cell with h) NC and i) MoNQDs/NC.

Given the equivalent criticality of Li^+^ evolution and kinetics for sulfur utilization, the regulatory mechanisms of catalysts on Li^+^ solvation shell architecture were further investigated. As depicted in Figure 3a, DFT calculations were conducted to determine adsorption energies of relevant species on surfaces, where the Li(DME)_4_^+^ was selected to simplify simulations given the prevalence of solvent‐separated ion pairs (SSIPs) in moderate electrolyte systems, while concurrently investigating TFSI^−^ anion adsorption behavior across substrates. Notably, both species exhibit significantly enhanced interaction strength on MoN compared to NC: adsorption energies are −3.8 eV versus −3.5 eV for Li(DME)_4_^+^ and −2.3 eV versus −1.0 eV for TFSI^−^, respectively. These enhanced interfacial interactions induce solvation shell reconstruction that facilitates Li(solvent)*_x_* ^+^ desolvation.^[^ ^30^, ^31^ ^]^ Furthermore, Raman spectroscopic analysis provides evidence for the structural reorganization of the solvation shell (Figure 3b). The solvated shell structures in the electrolyte can be categorized into three distinct types based on their coordination behaviors: SSIPs, contact ion pairs (CIPs), and aggregate ion pairs (AGGs). Notably, the combined proportion of CIPs and AGGs exhibits a strong correlation with the structural characteristics of the Li(solvent)*_x_* ^+^‐anion solvation shells. The electrolyte at the MoNQDs/NC catalyst interface exhibits a high proportion of CIPs and AGGs (74% and 19%, respectively), indicating that MoNQDs/NC can reconfigure the solvated shell structure and thus effectively weaken the ion‐dipole interactions between Li^+^ and solvent molecules (Figures 3c and S10). Consequently, this facilitates faster desolvation kinetics for Li(solvent)*_x_* ^+^ (Figure 3d). Then, DFT calculations were employed to simulate the adsorption behavior of Li on the materials. The results revealed that the adsorption energy of Li on NC reached −4.2 eV, significantly stronger than that on MoN (−1.2 eV) (Figure S11a). Such excessively strong adsorption energy can lead to irreversible reactions between Li and lithiophilic sites, resulting in the loss of lithiophilicity.^[^ ^32^ ^]^ Subsequently, the diffusion of Li on the material surface was further simulated. As revealed in Figure S11b, Li diffusion across the NC surface displayed a prohibitively high energy barrier of 3.5 eV. In striking contrast, the MoN surface demonstrated a substantially reduced diffusion barrier (0.4 eV), highlighting its capacity to enable rapid Li^+^ transport through significantly accelerated migration kinetics. To validate these simulation results, the performance of Li||Li symmetric batteries is tested. The cycling performance of cells at 0.5 mA cm^−2^ (0.5 mAh cm^−2^) was first tested (Figure 3e). The electrochemical measurements revealed that Li||Li symmetric cell with the MoNQDs/NC‐modified separator demonstrated significantly reduced overpotential and exceptional cycling stability over 1500 hours. Then, the rate performance of the cells was further evaluated at different current densities ranging from 0.5 to 5 mA cm^−2^. Compared to the cell with NC separator, the cell with MoNQDs/NC separator exhibited a smaller overpotential, suggesting enhanced uniform Li^+^ flux and stable Li plating/stripping performance (Figure S12a,b). Furthermore, finite element analysis based on COMSOL software was conducted to investigate the distribution of current density and the kinetic equilibrium of ion concentration. In the NC‐based system, the non‐uniform distribution of current density and Li^+^ flux leads to the accumulation of Li^+^ at the tips of Li protrusions, resulting in the vertical growth of sharp dendritic Li into the bulk electrolyte (Figure 3f). In contrast, in the MoNQDs/NC system, facilitated by the rapid electron‐Li^+^ transport, a higher Li^+^ concentration, uniform Li^+^ flux distribution, and homogeneous current density distribution are observed, thereby effectively suppressing the growth of Li dendrites (Figure 3g). These simulation results are in excellent agreement with the morphological characteristics of the Li anode shown in Figure 3h,i. In brief, a large number of Li dendrites formed on the Li metal surface of the NC battery, whereas the Li metal surface of the MoNQDs/NC battery remained relatively smooth. These results confirm that by weakening the ion‐dipole interaction in Li(solvent)*_x_* ^+^, MoNQDs/NC can promote uniform and rapid electron‐Li^+^ flux, thus exhibiting excellent interface stability.

**Figure 4:**
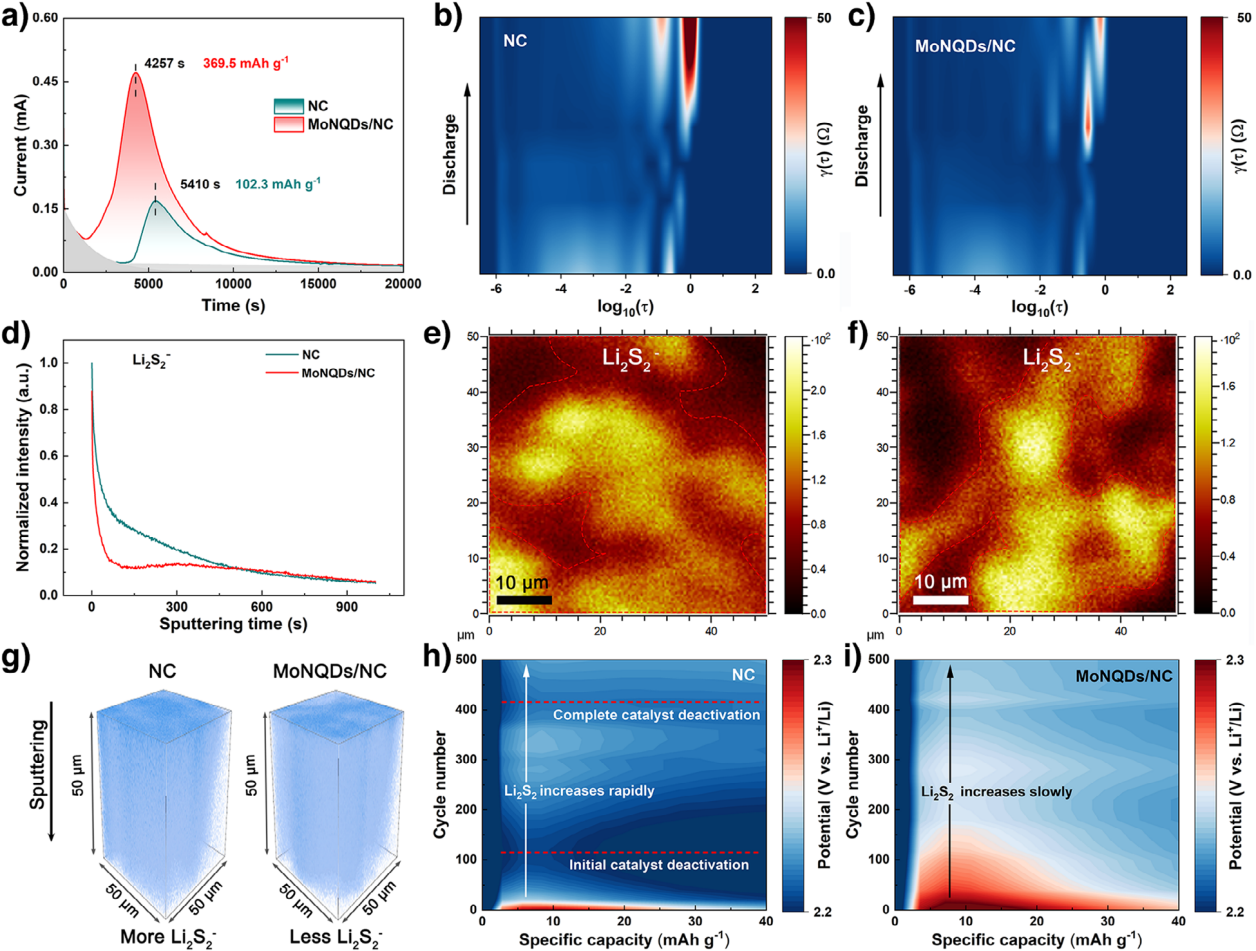
a) Precipitation profile of Li_2_S. Contour maps of DRT curves during discharge for the cells with b) NC and c) MoNQDs/NC. d) Depth sputtering profiles of Li_2_S_2_^−^ in the cathode by ToF‐SIMS. Mappings on the top surface of Li_2_S_2_^−^ for cells with e) NC and f) MoNQDs/NC. g) TOF‐SIMS 3D rendering Li_2_S_2_^−^ depth profile images. Contour maps of Li_2_S/Li_2_S_2_ dissolution during cycling at 0.2 C for the cells with h) NC and i) MoNQDs/NC.

Furthermore, the role of the materials on the kinetics of sulfur conversion was explored through additional precipitation and dissolution experiments of Li_2_S. The precipitation tests of Li_2_S showed that MoNQDs/NC composites exhibited the highest response current and an earlier peak in response current (4257 s) compared to NC (5410 s). And the calculated precipitation capacity of MoNQDs/NC reached 369.5 mAh g^−1^, far exceeding that of NC (102.3 mAh g^−1^), indicating that the MoNQDs/NC catalyst enhanced the liquid–solid reaction kinetics of LiPSs (Figure 4a). SEM images of the cathodes from the relevant Li_2_S precipitation cells are shown in Figure S13a,b. The cathode surface containing MoNQDs/NC exhibited a significant accumulation of solid discharge products, much more than that with NC, indicating an efficient reduction process of sulfur species. Quantitative analysis of Li_2_S dissolution kinetics unequivocally demonstrated the catalytic superiority of MoNQDs/NC, exhibiting a remarkable reduction in dissolution time by 58% (1346 s versus 3179 s) and a substantial increase in capacity utilization by 375% (556.5 mAh g^−1^ versus 117.1 mAh g^−1^) relative to the unmodified counterpart (Figure S14a). To analyze this process, DFT calculations were employed to simulate catalyst adsorption on Li_2_S. These simulations reveal significant stretching strain in Li_2_S under the dipole effect of MoN, manifested by elongation of Li─S bonds from 2.25 to 2.50 Å, indicating enhanced reaction activity (Figure S14b). Furthermore, the Li_2_S decomposition pathway was modeled. The results showed that compared to NC, the delithiation barrier for Li_2_S on MoN was significantly reduced (1.213 eV versus 0.406 eV), which is crucial for improving sulfur reutilization (Figure S14c).

To comprehensively evaluate the impact of catalysts on the kinetic performance of LSBs, complete full cells were assembled using different functional separators. Initially, the distribution of relaxation times (DRT) method was employed to analyze the impedance distribution. As shown in Figure 4b,c, charge transfer resistance (mid‐to‐high frequency region) governs the reaction kinetics during initial discharge, dictating the utilization efficiency of primary sulfur conversion. In contrast, diffusion impedance (low‐frequency region) dominates the overall cell resistance in mid‐late discharge stages, which correlates directly with the formation of solid sulfur species (Li_2_S_2_/Li_2_S). This phase corresponds to substantial phase transformation energy barriers for Li^+^ migration, representing the key rate‐limiting factor in sulfur reduction kinetics. Notably, compared to batteries with NC separator, batteries with MoNQDs/NC separator exhibited significantly smaller charge transfer resistance and diffusion resistance (indicated by lighter colors), marking an optimization in Li^+^ transport during SRR. Then, to probe the interfacial evolution and structural integrity of catalysts during electrochemical cycling, cells with NC and MoNQDs/NC separators were cycled at 1 C, with electrochemical impedance spectroscopy (EIS) acquired after 5, 10, and 20 cycles, respectively. As revealed by Figure S15, Nyquist plots for NC separators exhibit significant cycle‐dependent variation, specifically continuous expansion of the second semicircle (assigned to Li_2_S/Li_2_S_2_ accumulation^[^ ^33^ ^]^), indicating progressive interface degradation. In contrast, MoNQDs/NC separators maintain highly consistent plots across cycles with persistently stable charge‐transfer resistance, demonstrating their ion/dipole regulation mechanism sustains interfacial stability. Subsequently, the galvanostatic intermittent titration technique (GITT) was used to observe the kinetic processes of the batteries. The results indicated that during the discharge and charge processes, the MoNQDs/NC cell exhibited potential differences of 42 and 59 mV, respectively, which were lower compared to the 52 and 65 mV observed for the NC cell. This reduction in potential differences effectively decreased the internal resistance (Figure S16a,b). Additionally, Figure S17a shows the cyclic voltammetry (CV) curves at a sweep rate of 0.2 mV s^−1^. The results reveal that the MoNQDs/NC batteries not only exhibited higher current responses but also had smaller polarization potentials Δ*E*_L_ (0.15 V) and Δ*E*_H_ (0.48 V) compared to NC batteries (0.23 and 0.57 V) (Figure S17b). The Tafel plots derived from the CV curves are shown in Figure S17c–e, demonstrating that the batteries based on MoNQDs/NC exhibited lower Tafel slopes for all three redox peaks. These results indicate that MoNQDs/NC possesses excellent electrocatalytic activity, effectively regulating the redox reaction kinetics of sulfur species in LSBs.

The electrochemical evolution of sulfur species at the charge termination stage fundamentally governs the capacity fading and cycling performance in LSBs systems. Time‐of‐Flight Secondary Ion Mass Spectrometry (TOF‐SIMS) tests were conducted on the electrodes of batteries charged to 2.8 V after 10 cycles. Figure S18a,b present the depth sputtering profiles of S^−^, Li_2_S^−^, and Li_2_S_2_^−^ in the cathode of both cells. Post‐charge characterization clearly indicated the presence of residual Li_2_S/Li_2_S_2_ in the cathode, with Li_2_S_2_ being the predominant species. This observation strongly suggests that Li_2_S_2_, rather than Li_2_S, plays a determining role in the SOR. Further comparison of the depth sputtering profiles of Li_2_S_2_^−^ in cathodes, it was revealed that significantly less Li_2_S_2_^−^ was produced in the cathode of MoNQDs/NC batteries compared to NC batteries (Figure 4d). In addition, this conclusion is supported by the smaller Li_2_S_2_^−^ region in the ToF‐SIMS surface profile of the MoNQDs/NC system (Figure 4e,f). The 3D construction images of Li_2_S_2_^−^ in the cathode also demonstrated less Li_2_S_2_ distribution in MoNQDs/NC batteries, suggesting high utilization of Li_2_S_2_ by MoNQDs/NC (Figure 4g). Moreover, the less distribution of Li_2_S^−^ in the anode of the MoNQDs/NC battery demonstrates that the shuttle effect is suppressed (Figure S19a,b). To further validate the catalytic effect of the material on the decomposition kinetics of Li_2_S_2_, the charging process of the cycled battery within the potential range of 3.0 to 3.8 V was analyzed. The response current at 3.53 V corresponds to electrolyte decomposition.^[^ ^34^ ^]^ It is noteworthy that at 3.56 V, NC batteries exhibited a notable peak in response current, attributable to the reactivation of deactivated Li_2_S_2_. In comparison, the MoNQDs/NC battery exhibited virtually no additional current peak, indicating a lower content of Li_2_S_2_ in the cathode (Figure S20). This observation confirms that the decomposition kinetics of the Li_2_S_2_ have also been enhanced.

The impact of the presence of Li_2_S_2_ on the cycling performance of the battery was then evaluated. As shown in Figure S21, the battery utilizing the MoNQDs/NC separator maintained a stable capacity of 667 mAh g^−1^ after 500 cycles at 0.2 C. In contrast, the battery with NC separator experienced a rapid capacity decline around the 350th cycle, ultimately reaching only 300 mAh g^−1^. Analysis of the dissolution process of solid‐state products Li_2_S/Li_2_S_2_ during cycling for both types of batteries is presented in Figure 4h,i. As the number of cycles increased, the dissolution peak potentials of Li_2_S/Li_2_S_2_ for both batteries initially decreased to varying degrees. This may be due to the accumulation of Li_2_S_2_ in the cathode and the disproportionation decomposition of Li_2_S_2_ to generate a significant amount of liquid LiPSs. The generated soluble LiPSs can further undergo chemical neutralization reactions with Li_2_S, thereby reducing the peak potential of the dissolution process. Notably, in Figure 4h, the peak potential of the NC battery begins to gradually increase after about 100 cycles. This may be due to the initial coverage of the catalyst by Li_2_S_2_, leading to a decrease in its ability to catalyze the oxidation process of Li_2_S/Li_2_S_2_.^[^ ^35^ ^]^ By the 400th cycle, the catalyst is completely deactivated, which corresponds to the sharp decline in the cycling performance of the NC battery. In contrast, the dissolution peak potential of Li_2_S/Li_2_S_2_ for the battery with the MoNQDs/NC separator decreases slowly and does not exhibit a significant re‐increase. This indicates that most of the inert Li_2_S_2_ has been utilized and has not caused catalyst deactivation due to coverage (Figure 4i). These results demonstrate that MoNQDs/NC can effectively regulate the evolution of Li_2_S_2_, thereby enabling the efficient utilization of Li_2_S_2_ during the sulfur redox reactions.

**Figure 5:**
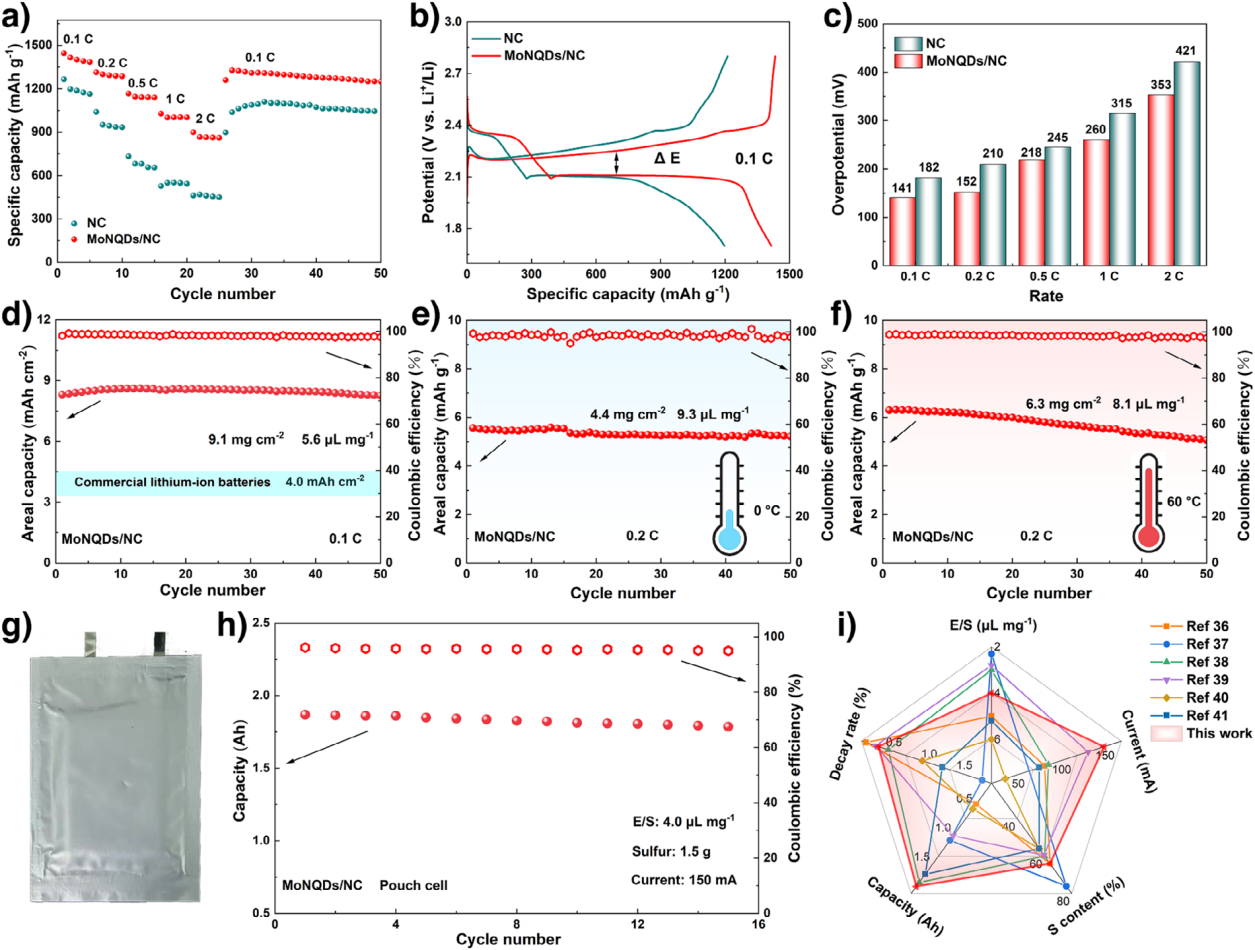
a) Rate performance and b) the corresponding GCD curves at 0.1 C. c) Overpotential at different rates. Cycling performance of high‐sulfur‐loading batteries at d) room temperature, e) low temperature, and f) high temperature, respectively. g) Optical photograph of an Ah‐level MoNQDs/NC pouch cell. h) Cycling performance of the assembled pouch cell. i) Performance comparison between this work and the recently reported pouch cells.

Subsequently, the electrochemical performance of LSBs with different separators was evaluated under various conditions. Initially, the rate performance of the batteries was investigated. As shown in Figure 5a, as the rate increased from 0.1 to 2 C, the capacity advantage of cells with the MoNQDs/NC separator became progressively more evident compared to those utilizing the NC separator (898.7 mAh g^−1^ versus 462.6 mAh g^−1^). The galvanostatic charge‐discharge (GCD) curves of MoNQDs/NC cell at different rates all exhibited two distinct charge–discharge platforms, corresponding to the rapid reaction kinetics during the solid–liquid–solid transformation of sulfur species (Figures 5b and S22a,b). The modified cell exhibited the lower overpotentials in Figure 5c, which also confirmed that MoNQDs/NC facilitated the enhancement of sulfur redox reversibility. Additionally, the MoNQDs/NC‐based cell exhibited a significantly lower capacity decay rate (3.4%) compared to the NC‐based cell (10.5%) after a 72 h resting period, demonstrating enhanced resistance to self‐discharge (Figure S23). The cycling performance of batteries with high sulfur loading was further explored. As shown in Figure 5d, at a sulfur loading of 9.1 mg cm^−2^, the battery achieved a high initial areal capacity of 8.3 mAh cm^−2^, far exceeding that of current commercial Li‐ion batteries (∼4.0 mAh cm^−2^). Notably, even after 50 cycles, the capacity retention rate of the high‐sulfur‐loading battery reached 99.4%. The high‐sulfur‐loading battery with the MoNQDs/NC separator still demonstrates significant advantages compared to recently reported systems (Table S1). In order to explore the adaptability of the batteries to temperature, the temperature range for batteries testing was broadened. At a low temperature of 0 °C, the battery exhibited a capacity decay rate of only 0.018% per cycle after 100 cycles at 1 C (Figure S24). Subsequently, the adaptability of high‐sulfur‐loading batteries over a wide temperature range was explored. The electrochemical cycling performance of high‐sulfur‐loading cathodes (4.4 and 6.3 mg cm^−2^) was systematically evaluated at extreme temperatures (0 and 60 °C). Remarkably, the modified cells under these conditions all demonstrated excellent capacity retention, with areal capacities above 5.0 mAh cm^−2^ after 50 cycles at 0.2 C (Figure 5e,f). To further evaluate the practical applicability of the MoNQDs/NC interlayer, an Ah‐level multilayer pouch cell was assembled (Figure 5g). The cell featured a total sulfur loading of 1.5 g and an E/S ratio of ≈4.0 µL mg^−1^. Notably, the pouch cell achieved a discharge capacity of 1.87 Ah at 150 mA and retained a capacity of 1.79 Ah after 15 cycles (Figure 5h). Compared with state‐of‐the‐art LSBs reported in recent literature, the MoNQDs/NC‐modified pouch cell demonstrated exceptional overall performance (Figure 5i).^[^ ^36^, ^37^, ^38^, ^39^, ^40^, ^41^ ^]^ These results provide support for the feasibility of catalytic strategies that cooperatively modulate ion/dipole interactions.

## Conclusion

In summary, a catalytic strategy based on ion‐dipole co‐regulation at catalytic interfaces has been proposed by constructing MoNQDs/NC as functional catalysts for concept validation. The combination of DFT calculations, COMSOL multi‐physics simulations, and in situ/ex situ electrochemical analyses systematically demonstrates that MoNQDs/NC effectively weakens ion‐dipole interactions within the solvation shell structure, and the periodic Mo–N atomic arrangement in quantum dots creates a localized dipole field that induces strong dipole‐dipole interactions with Li_2_S_2_. Consequently, batteries with MoNQDs/NC separators maintained a high areal capacity close to 8.3 mAh cm^−2^ at a high sulfur loading of 9.1 mg cm^−2^ with a low capacity decay rate of 0.012% per cycle. More importantly, at ambient temperatures of 0 and 60 °C, the areal capacity of high‐sulfur‐loading batteries still exceeded 5.0 mAh cm^−2^ after 50 cycles at 0.2 C. Furthermore, the pouch cell employing lean electrolyte (E/S ratio = 4.0 µL mg^−1^) achieved a discharge capacity of 1.87 Ah at 150 mA, demonstrating both high energy density and exceptional cycling stability. The electrocatalyst synergistic modification strategy of ion‐dipole interaction provides a new direction for the development of practical LSBs.

## Supporting Information

The authors have cited additional references within the Supporting Information.^[^ ^42^, ^43^, ^44^, ^45^, ^46^, ^47^, ^48^, ^49^, ^50^, ^51^ ^]^

## Conflict of Interests

The authors declare no conflict of interest.

## Supporting information



Supporting Information

## Data Availability

The data that support the findings of this study are available from the corresponding author upon reasonable request.

## References

[1] Z. W. Seh , Y. Sun , Q. Zhang , Y. Cui , Chem. Soc. Rev. 2016, 45, 5605–5634. 27460222 10.1039/c5cs00410a

[2] J. Shen , Z. Liang , T. Gu , Z. Sun , Y. Wu , X. Liu , J. Liu , X. Zhang , J. Liu , L. Shen , M. Zhu , J. Liu , Energy Environ. Sci. 2024, 17, 6034.

[3] H.‐J. Peng , Z.‐W. Zhang , J.‐Q. Huang , G. Zhang , J. Xie , W.‐T. Xu , J.‐L. Shi , X. Chen , X.‐B. Cheng , Q. Zhang , Adv. Mater. 2016, 28, 9551–9558. 27629655 10.1002/adma.201603401

[4] S. Li , B. Jin , X. Zhai , H. Li , Q. Jiang , ChemistrySelect 2018, 3, 2245–2260.

[5] J. Wang , W.‐Q. Han , Adv. Funct. Mater. 2022, 32 2107166.

[6] Z. Liang , J. Shen , X. Xu , F. Li , J. Liu , B. Yuan , Y. Yu , M. Zhu , Adv. Mater. 2022, 34, 2200102. 10.1002/adma.202200102 35238103

[7] J. Wang , J. Zhang , Y. Zhang , H. Li , P. Chen , C. You , M. Liu , H. Lin , S. Passerini , Adv. Mater. 2024, 36, 2402792. 10.1002/adma.202402792 38616764

[8] L. Ma , Y. Wang , Z. Wang , J. Wang , Y. Cheng , J. Wu , B. Peng , J. Xu , W. Zhang , Z. Jin , ACS Nano 2023, 17, 11527–11536. 37288710 10.1021/acsnano.3c01469

[9] H. Pan , X. Wei , W. A. Henderson , Y. Shao , J. Chen , P. Bhattacharya , J. Xiao , J. Liu , Adv. Energy Mater. 2015, 5, 1500113.

[10] Y. Liu , Y. Elias , J. Meng , D. Aurbach , R. Zou , D. Xia , Q. Pang , Joule 2021, 5, 2323–2364.

[11] L. Chen , J. Lai , X. Guan , H. Zou , J. Liu , L. Peng , J. Wang , Y.‐P. Cai , Q. Zheng , Angew. Chem. Int. Ed. 2025, 64, e202423046. 10.1002/anie.202423046 39815714

[12] J. Shen , X. Xu , J. Liu , Z. Wang , S. Zuo , Z. Liu , D. Zhang , J. Liu , M. Zhu , Adv. Energy Mater. 2021, 11, 2100673.

[13] Y. Zhong , Q. Wang , S.‐M. Bak , S. Hwang , Y. Du , H. Wang , J. Am. Chem. Soc. 2023, 145, 7390–7396. 36952313 10.1021/jacs.2c13776

[14] A. Berger , A. T. S. Freiberg , A. Siebel , R. Thomas , M. U. M. Patel , M. Tromp , H. A. Gasteiger , Y. Gorlin , J. Electrochem. Soc. 2018, 165, A1288–A1296.

[15] J. Conder , R. Bouchet , S. Trabesinger , C. Marino , L. Gubler , C. Villevieille , Nat. Energy 2017, 2, 17069.

[16] Y. Luo , Z. Fang , S. Duan , H. Wu , H. Liu , Y. Zhao , K. Wang , Q. Li , S. Fan , Z. Zheng , W. Duan , Y. Zhang , J. Wang , Angew. Chem. Int. Ed. 2023, 62, e202215802. 10.1002/anie.202215802 36650422

[17] R. Yan , Z. Zhao , M. Cheng , Z. Yang , C. Cheng , X. Liu , B. Yin , S. Li , Angew. Chem. Int. Ed. 2023, 62, e202215414. 10.1002/anie.202215414 PMC10107143 36321878

[18] M. Liu , Z. Wu , S. Liu , T. Guo , P. Chen , X. Cao , S. Pan , T. Zhou , L. Pompizii , M. Najafov , A. Coskun , Y. Fu , Angew. Chem. Int. Ed. 2025, 64, e202417624. 10.1002/anie.202417624 39345165

[19] X. Zhang , X. Li , Y. Zhang , X. Li , Q. Guan , J. Wang , Z. Zhuang , Q. Zhuang , X. Cheng , H. Liu , J. Zhang , C. Shen , H. Lin , Y. Wang , L. Zhan , L. Ling , Adv. Funct. Mater. 2023, 33, 2302624.

[20] J. Wang , H. Liu , J. Zhang , Q. Xiao , C. Wang , Y. Zhang , M. Liu , Q. Kang , L. Jia , D. Wang , Q. Li , W. Duan , H. Adenusi , S. Passerini , Y. Zhang , H. Lin , Energy Storage Mater. 2024, 67, 103289.

[21] F. Ma , K. Srinivas , X. Zhang , Z. Zhang , Y. Wu , D. Liu , W. Zhang , Q. Wu , Y. Chen , Adv. Funct. Mater. 2022, 32, 2206113.

[22] L. Gao , B. Jing , X. Wang , Q. Cao , Z. Ma , Mater. Today Chem. 2024, 42, 102437.

[23] Z.‐L. Xu , S. Lin , N. Onofrio , L. Zhou , F. Shi , W. Lu , K. Kang , Q. Zhang , S. P. Lau , Nat. Commun. 2018, 9, 4164. 30301957 10.1038/s41467-018-06629-9 PMC6177446

[24] Y. Kong , L. Wang , M. Mamoor , B. Wang , G. Qu , Z. Jing , Y. Pang , F. Wang , X. Yang , D. Wang , L. Xu , Adv. Mater. 2024, 36, 2310143. 10.1002/adma.202310143 38134811

[25] W. Xia , Y. Chen , M. Han , X. Wu , H. Yang , K. Fu , M. Chen , X. Wang , H. Shu , Adv. Funct. Mater. 2024, 34, 2400262.

[26] Q. Chen , T. Li , H. Huang , W. Wang , Z. Yu , Q. Liu , L. Liu , Nano Energy 2025, 133, 110509.

[27] D. Zhang , T. Duan , Y. Luo , S. Liu , W. Zhang , Y. He , K. Zhu , L. Huang , Y. Yang , R. Yu , X. Yang , H. Shu , Y. Pei , X. Wang , M. Chen , Adv. Funct. Mater. 2023, 33, 2306578.

[28] C. Zhao , G.‐L. Xu , Z. Yu , L. Zhang , I. Hwang , Y.‐X. Mo , Y. Ren , L. Cheng , C.‐J. Sun , Y. Ren , X. Zuo , J.‐T. Li , S.‐G. Sun , K. Amine , T. Zhao , Nat. Nanotechnol. 2021, 16, 166–173. 33230316 10.1038/s41565-020-00797-w

[29] Z. Liu , M. Chen , D. Zhou , Z. Xiao , Adv. Funct. Mater. 2023, 33, 2306321.

[30] F. Zhu , J. Wang , Y. Zhang , H. Tu , X. Xia , J. Zhang , H. He , H. Lin , M. Liu , Adv. Mater. 2025, 37, 2411601. 39679840 10.1002/adma.202411601 PMC11795707

[31] Z. Zhang , J. Wang , H. Qin , B. Zhang , H. Lin , W. Zheng , D. Wang , X. Ji , X. Ou , ACS Nano 2024, 18, 2250–2260. 38180905 10.1021/acsnano.3c09849

[32] Z. Yang , Y. Dang , P. Zhai , Y. Wei , Q. Chen , J. Zuo , X. Gu , Y. Yao , X. Wang , F. Zhao , J. Wang , S. Yang , P. Tang , Y. Gong , Adv. Energy Mater. 2022, 12, 2103368.

[33] C. Zhang , R. Du , J. J. Biendicho , M. Yi , K. Xiao , D. Yang , T. Zhang , X. Wang , J. Arbiol , J. Llorca , Y. Zhou , J. R. Morante , A. Cabot , Adv. Energy Mater. 2021, 11, 2100432.

[34] C. Zha , S. Wang , C. Liu , Y. Zhao , B. He , C. Lyu , J. Li , S. Ji , S. Chen , K. S. Hui , K. N. Hui , Energy Storage Mater. 2022, 47, 79–86.

[35] S.‐Y. Lang , Y. Shi , Y.‐G. Guo , D. Wang , R. Wen , L.‐J. Wan , Angew. Chem. Int. Ed. 2016, 55, 15835–15839. 10.1002/anie.201608730 27860060

[36] R. Yang , Y. Chen , Y. Pan , M. Kim , H. Liu , C. K. W. Lee , Y. Huang , A. Tang , F. Tu , T. Li , M. G. Li , Nat. Commun. 2025, 16, 2386. 40064904 10.1038/s41467-025-57755-0 PMC11894212

[37] S. Li , Z. Chen , J. Chen , X. Luo , X. Qiu , Y. Qian , Adv. Energy Mater. 2025, 15, 2405461.

[38] J. Wang , X. Zhang , X. Wang , J. Liu , S. Li , Y. Nie , K. Zong , X. Zhang , H. Meng , M. Jin , L. Yang , X. Wang , Z. Chen , Adv. Energy Mater. 2024, 14, 2402072.

[39] M. Li , H. Liu , Z. Cheng , J. He , H. Li , L. Zhang , T. Liu , X. Wang , P. Wang , Z. Liu , G. Cui , Adv. Energy Mater. 2025, 15, 2405766.

[40] L. Ji , D. Yang , J. Xue , M. Jia , T. Wu , Q. Zhuang , Y. Zhang , J. Liu , Y. Zhang , Adv. Energy Mater. 2025, 15, 2404738.

[41] Gao , M. Zhang , Z. Han , X. Xiao , X. Wu , Z. Piao , Z. Lao , L. Nie , S. Wang , G. Zhou , Adv. Mater. 2024, 36, 2303610. 10.1002/adma.202303610 37500064

[42] G. Kresse , J. Furthmüller , Phys. Rev. B 1996, 54, 11169–11186. 10.1103/physrevb.54.11169 9984901

[43] G. Kresse , J. Furthmüller , Comput. Mater. Sci. 1996, 6, 15–50.

[44] J. P. Perdew , K. Burke , M. Ernzerhof , Phys. Rev. Lett. 1996, 77, 3865–3868. 10062328 10.1103/PhysRevLett.77.3865

[45] P. E. Blöchl , Phys. Rev. B 1994, 50, 17953. 10.1103/physrevb.50.17953 9976227

[46] S. Grimme , J. Antony , S. Ehrlich , H. Krieg , J. Chem. Phys. 2010, 132, 154104. 20423165 10.1063/1.3382344

[47] G. Henkelman , B. P. Uberuaga , H. Jónsson , J. Chem. Phys. 2000, 113, 9901–9904.

[48] Z.‐H. Luo , M. Zheng , M.‐X. Zhou , X.‐T. Sheng , X.‐L. Chen , J.‐J. Shao , T.‐S. Wang , G. Zhou , Adv. Mater. 2025, 37, 2417321. 10.1002/adma.202417321 39846826

[49] J. Pu , S. Fan , Z. Shen , J. Yin , Y. Tan , K. Zhang , B. Wu , G. Hong , Y. Yao , Adv. Funct. Mater. 2025, 35, 2424215.

[50] K. Wang , Y. Wang , J. Wang , H. Wang , C. Ding , Z. Zheng , Y. Liu , Z. Luo , Y. Ding , Adv. Funct. Mater. 2025, 35, 2422689.

[51] R. Qi , L. Zhao , P. Liu , Y. Zhen , X. Fu , X. Li , Y. Cui , T. Cai , Z. Yan , Q. Xue , W. Xing , Energy Storage Mater. 2025, 75, 104065.

