# Supplementary material for: Modulating Ion‐Dipole and Dipole–Dipole Interactions for Stable Wide‐Temperature‐Range Lithium–Sulfur Batteries Enabled by Quantum‐Dot Catalysts
Source: Angew Chem Int Ed Engl. 2025 Aug 5;64(39):e202512168. doi: 10.1002/anie.202512168 (PMC12455398; doi:10.1002/anie.202512168)
Supplement: Supplementary file 1 — Supporting Information [file ANIE-64-e202512168-s001.docx]

**Supporting In****formation**

**Modulating Ion-Dipole and Dipole-Dipole Interactions for Stable Wide-Temperature-Range Lithium-Sulfur Batteries Enabled by Quantum-Dot Catalysts**

Yongqian He^[a]^, Duanfeng Xiong^[c]^, Manfang Chen^[a]^*, Wanqi Zhang^[a]^, Sisi Liu^[a]^, Yongjie Ye^[a]^, Mengqing Wang^[a]^, Ying Chen^[a]^, Qin Tang^[a]^, Xuewen Peng^[a]^, Caixiang Wang^[a]^, Hongyang Zhan^[a]^, Hong Liu^[a]^, Min Liu^[b]^*, Jincang Su^[c]^*, Hongbo Shu^[a]^, Jian Wang^[d]^*, Xianyou Wang^[a]^

[a] National Base for International Science & Technology Cooperation of New Energy Equipment, Energy Storage Materials and Devices, National Local Joint Engineering Laboratory for Key Materials of New Energy Storage Battery, Hunan Province Key Laboratory of Electrochemical Energy Storage & Conversion, School of Chemistry, Xiangtan University, Xiangtan 411105, Hunan, China

E-mail: [mfchen@xtu.edu.cn](mailto:mfchen@xtu.edu.cn)

[b] College of New Energy, Ningbo University of Technology, Ningbo, Zhejiang 315336, China

E-mail: [liumin@nbut.edu.cn](mailto:liumin@nbut.edu.cn)

[c] School of Materials Science and Engineering, Xiangtan University, Xiangtan 411105, China

E-mail: [sujc@xtu.edu.cn](mailto:sujc@xtu.edu.cn)

[d] Helmholtz Institute Ulm (HIU), Ulm D89081, Germany

Karlsruhe Institute of Technology (KIT), Karlsruhe D-76021, Germany

E-mail: [jian.wang@kit.edu](mailto:jian.wang@kit.edu)

**Section SI. Experimental Section**

**1.1 Synthesis of NC and** **MoNQDs/NC**

3 g melamine and 1 g (NH_4_)_6_Mo_7_O_24_·4H_2_O (≥ 99 wt%, Macklin Biochemical) were dissolved in 50 mL deionized water, which was then sealed in a reactor, heated to 200 ℃ and held for 24 hours. After the reaction, the resulting product was filtered to obtain a white powder, which was then dried in a drying oven at 60 ℃ for 12 hours. The powder was then placed downstream in a tube furnace, and 2 g of hexamethylenetetramine was placed upstream in the tube furnace and held at 800 ℃ for 2 hours at a heating rate of 5 ℃ min^-1^ under an Ar atmosphere containing 5% H_2_. The resulting powder was MoNQDs/NC. NC was prepared without the addition of (NH_4_)_6_Mo_7_O_24_·4H_2_O and other conditions were not changed.

**1.2 Synthesis of sulfur cathode**

In an onyx mortar, 0.1 g of carbon nanotubes (CNT) and 0.4 g of sulfur (S) powder were mixed, and then the mixture was collected and heated in a closed vessel at 155 ℃ for 12 h to obtain the CNT/S powder. The CNT/S, Super P (SP) and polyvinylidene fluoride (PVDF) were mixed homogeneously in a ratio of 8:1:1, respectively, using N-methylpyrrolidone (NMP) as the solvent. The mixture was then coated onto Al foil and dried in vacuum at 55 ℃ for 24 hours, which resulted in the formation of CNT/S cathodes with a sulfur mass loading of 1.2 mg cm^–2^ or a high sulfur mass loading of 4.4~9.1 mg cm^–2^.

**1.3 Material characterizations**

The micro-morphology of the samples were analyzed by transmission electron microscope (TEM, FEI TF20, Netherlands). Energy-dispersive X-ray spectroscopy (EDX) was employed to assess the elemental distribution of the sample. The crystal structure of the synthesized samples were determined via an X’Pert Pro MPD X-ray diffractometer (XRD, Panalytical, Netherlands) with Cu-Kα radiation over the 2θ range of 10-80°. X-ray photoelectron spectrum (XPS, PHI 5000 Versa Probe, Japan) was utilized to characterize the compositions of the samples. Raman spectra (DXR Raman Microscope) was used to distinguish anion structures at the electrolyte/catalyst interface. Time–of–flight secondary ion mass spectroscopy (ToF–SIMS) analyses were performed with Cs^-^ sputter condition (5 keV, 35 nA).

**1.4 Electrochemical measurements**

The functional material, SP, and PVDF were mixed homogeneously in a ratio of 8:1:1 respectively, using NMP as the solvent. The mixture was then coated onto Celgard polypropylene (PP) film and dried before being cut into 19 mm diameter separators. In an Ar-filled glove box, a CR2025 coin cell was assembled with the sulfur cathode, separator, anode (Li metal) and electrolyte, where the amount of electrolyte was 30~40 μL. The electrolyte consisted of a mixture of 1,3-dioxolane/1,2-dimethoxyethane (DOL/DME, 1:1 volume) containing 1wt.% LiNO_3_ and 1 mol L^–1^ Li bis (trifluoromethane sulfonyl) imide (LiTFSI).

Galvanostatic charge/discharge measurements were conducted using a battery testing instrument (Neware CT-4800T) within a potential window of 1.7-2.8 V at room temperature to collect data at different C-rates (1 C = 1675 mA g^–1^). Cyclic voltammograms (CV) curves were obtained with a sweep rate of 0.2 mV s^–1^ in potential ranges of 1.7-2.8 V using an electrochemical workstation (CHI 660E).

**1.5 Assembly of the pouch cell**

Multilayer pouch cells were assembled using double-sided electrodes (5.0 cm × 10.0 cm). The sulfur-based cathode was prepared by coating both sides of the aluminum foil collector with a sulfur-containing slurry, with a sulfur loading of approximately 5.0 mg cm^-2^. The Li foil anode (thickness: 0.1 mm) was cut to dimensions of 5.5 cm × 10.5 cm. The modified separator, Li anode, and sulfur cathode were assembled in an Ar-filled glove box using aluminum-plastic film packaging. The electrolyte was injected at an E/S ratio of ≈ 4.0 μL mg^-1^, followed by vacuum sealing.

**1.6 In-situ Ultraviolet-visible (UV-vis) spectra measurement**

The preparation of sulfur-containing cathode materials followed the same procedure as before, and the materials were then cut into rectangles measuring 0.8 cm × 1.5 cm. The sulfur area mass loading on the electrode was approximately 4.5 mg cm^−2^. In an Ar full-filled glove box, the in-situ UV-vis cells were assembled using NC/S, MoNQDs/NC/S electrode as the cathode, and Li metal as the anode. The assembly was carried out using a custom-made in-situ cuvette purchased from Tianjin Aida Hengsheng Technology Development Co., LTD. The assembled cells were further sealed with Li–S electrolyte (3 mL). The in-situ cuvette cell was galvanostatically discharged at 0.1 C using an electrochemical workstation. UV-vis spectra were recorded every 15 minutes within a wavelength range of 450-650 nm from the beginning of the discharge until the end of the discharge for the batteries.

**1.7 Measurements of the** **galvanostatic intermittent titration technique (GITT)**

A protocol involving a current pulse at 0.1 C for 30 minutes followed by 60 minutes of rest was utilized.

**1.8 Preparation of Li_2_S_8_ and measurements of Li_2_S precipitation (dissolution)**

Li_2_S_8_ solution (0.1 mol L^–1^) was prepared by dissolving the stoichiometric amounts of S and Li_2_S with a molar ratio of 7:1 in DME/DOL (v/v = 1:1) solution. The coin cell was assembled by using the carbon paper (CP) loaded with different materials as the cathode, Celgard 2400 PP as separator, the Li metal as the anode. And the Li_2_S_8_ (25 μL) was dropped onto the cathode, and conventional LiTFSI electrolyte (25 μL) was added to the anode side. For Li_2_S precipitation (dissolution), the battery were discharged galvanostatically to 2.06 V (1.7 V) at 0.112 mA, and then discharged (charged) potentiostatically at 2.05 V (2.4 V) for 80000 seconds. The precipitation (dissolution) capacity of Li_2_S can be calculated through Faraday’s Law.

**1.9 Preparation of** **Li_2_S_6_**

Li_2_S_6_ solution (0.1 mol L^–1^) was prepared by dissolving the stoichiometric amounts of S and Li_2_S with a molar ratio of 5:1 in DME/DOL (v/v = 1:1) solution.

**1.10 COMSOL Multiphysics simulations**

Finite element simulations were performed using COMSOL Multiphysics within a 6 μm × 6 μm rectangular domain to model Li electrodeposition. The coupled model simultaneously solves for the electric field (current density distribution), concentration field (Li^+^ distribution), and phase field (Li deposition morphology). A transient solver with mesh refinement at the electrode boundary was employed to ensure accurate resolution of the interfacial evolution.

**1.11 Density functional theory calculations**

The first-principal calculations were conducted utilizing spin-polarized density functional theory (DFT) within the Vienna ab initio simulation package (VASP 6.1).^[1]^ The electron exchange correlation was described by the gradient-corrected Perdew-Burke-Ernzerh (GGA-PBE) functional.^[2]^ Ion-electron interaction was described by the projector augmented-wave (PAW) method.^[3]^ The cutoff energy was established at 500 eV, with the total energy and force convergence for geometric optimization set at 10^-5^ eV and 0.01 eV/Å, respectively. For structural relaxation, a grid of 5 × 5 × 1 Gamma-centered k-points was employed. A vacuum layer of 40 Å in the z-direction was utilized, and a semi-empirical correction of DFT-D3^[4]^ was applied to consider the van der Waals forces interaction. The decomposition barrier of Li_2_S and Li_2_S_2_ and the diffusion energy barrier of Li^+^ were determined by climbing-image nudged elastic band (CI-NEB)^[5]^ method.

**Section SII. Supporting Figures and Table**
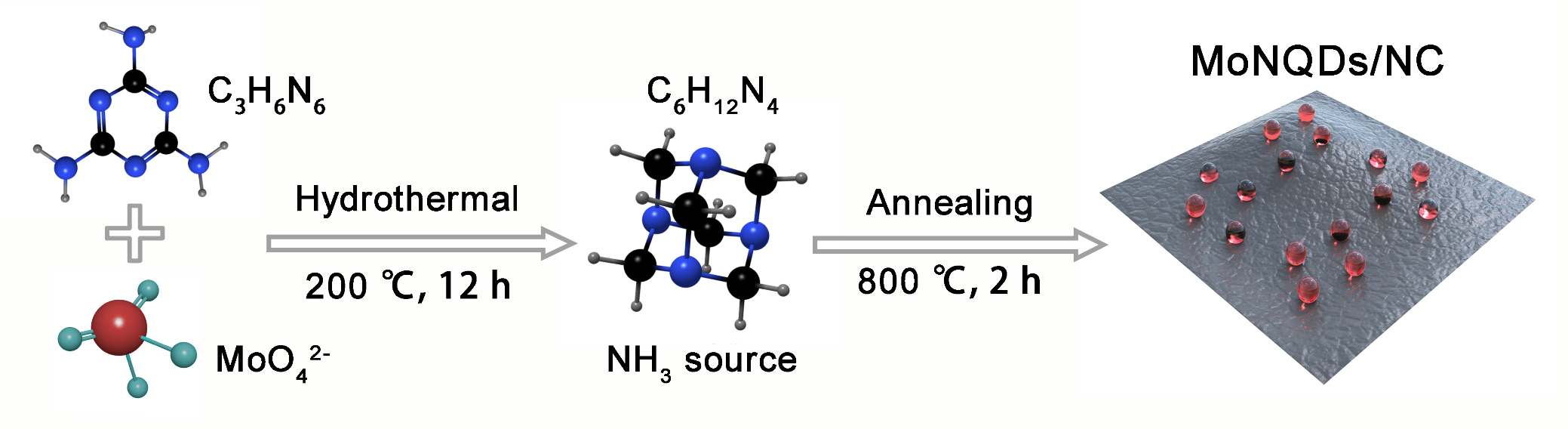


**Figure S1.** The synthesis diagram of MoNQDs/NC.


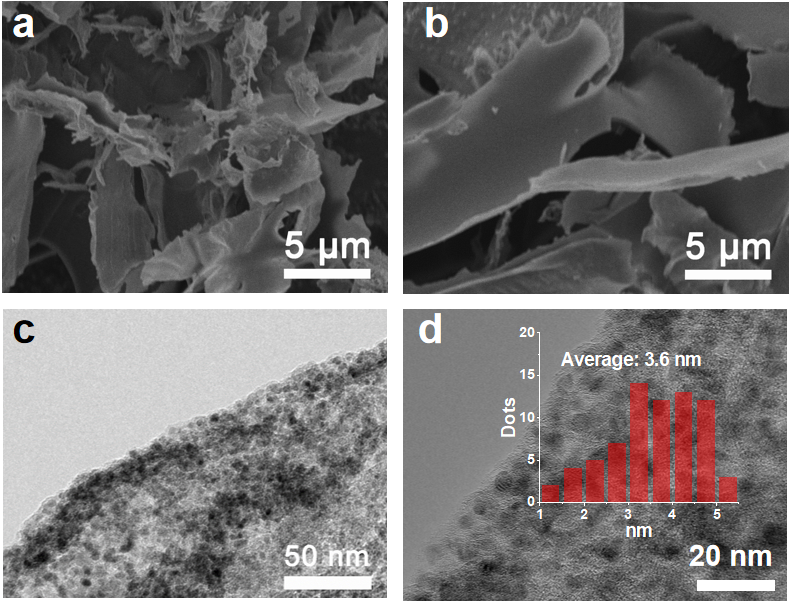


**Figure S2.** SEM images of a) NC and b) MoNQDs/NC. c) TEM image of MoNQDs/NC. HRTEM image of MoNQDs/NC (inset shows the corresponding particle size distribution).


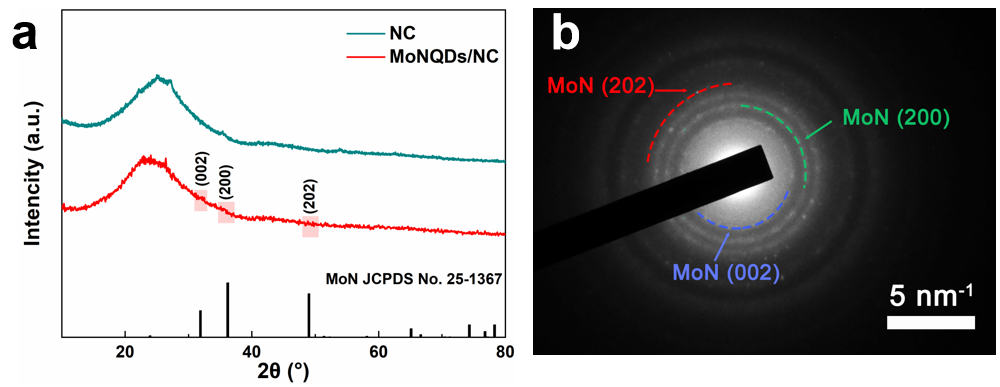


**Figure S3.** a) XRD spectra of materials. b) SAED image of MoNQDs/NC.


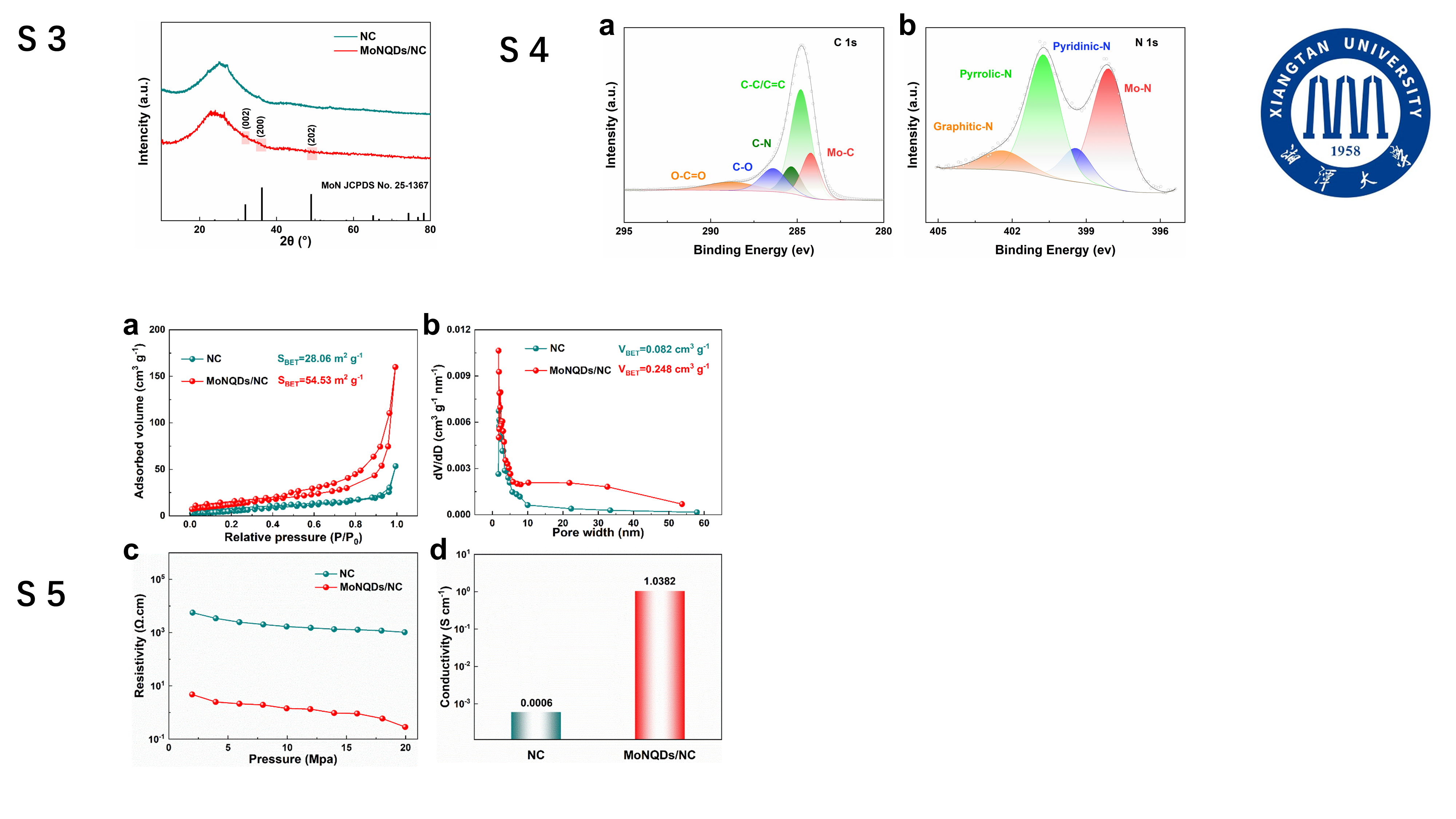


**Figure S4.** XPS spectra of a) C 1s and b) N 1s of MoNQDs/NC.


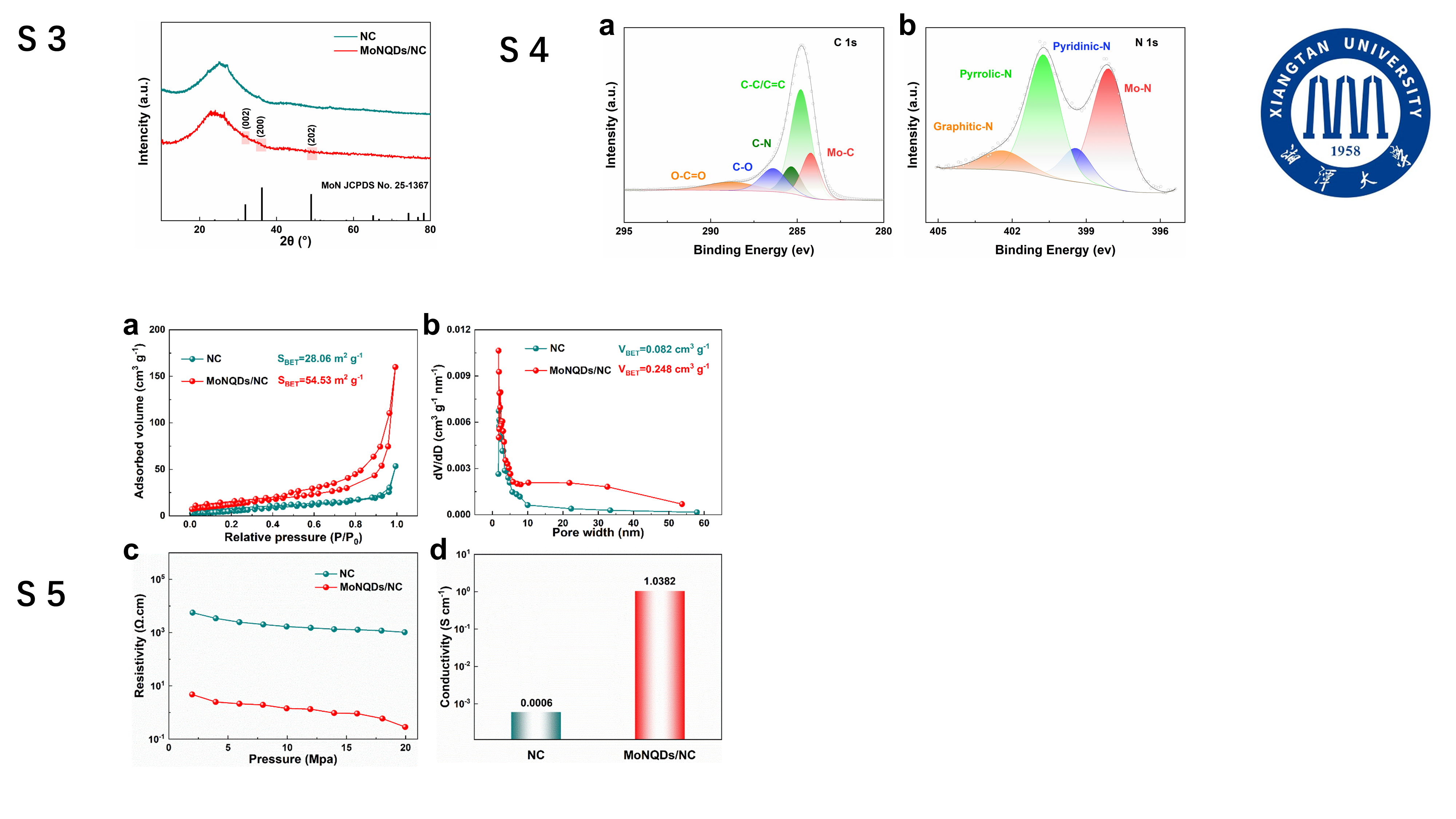


**Figure S5.** a) N_2_ adsorption-desorption isotherms and b) pore size distribution of materials. c) resistivity and d) conductivity of materials.


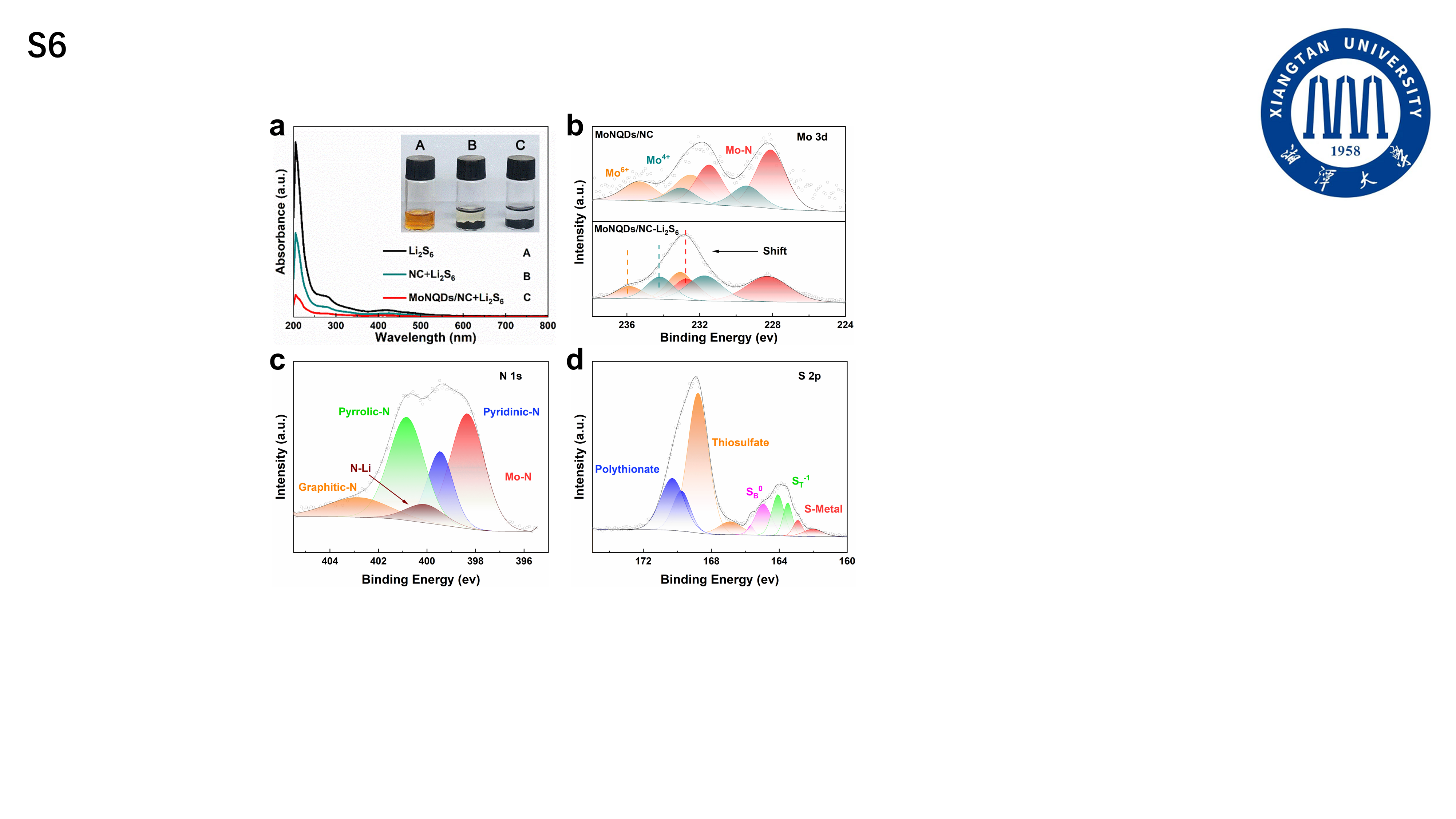


**Figure S6.** a) UV–vis spectra and optical photograph of Li_2_S_6_ solutions after interacting with NC and MoNQDs/NC. b) XPS spectra of Mo 3d of MoNQDs/NC before and after adsorption of Li_2_S_6_. XPS spectra of c) N 1s and d) S 2P of MoNQDs/NC after adsorption of Li_2_S_6_.

**
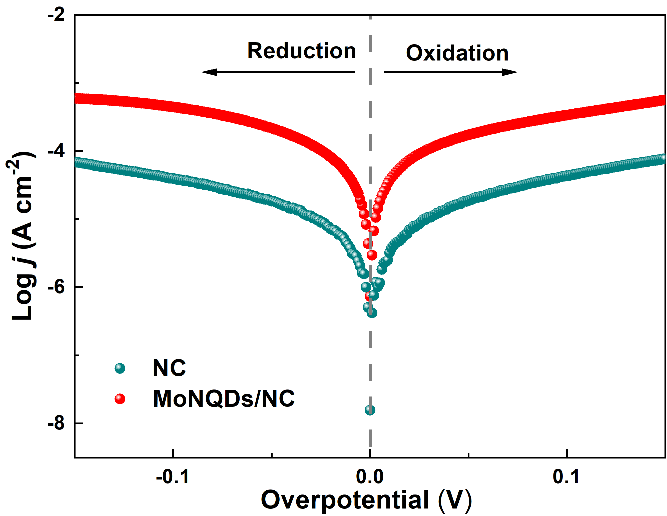
**

**Figure S7.** Tafel plots based on Li_2_S_6_ symmetric cells.


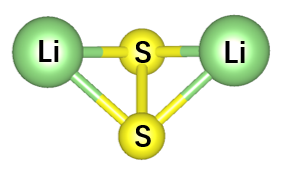


**Figure S8.** Model of Li_2_S_2_.


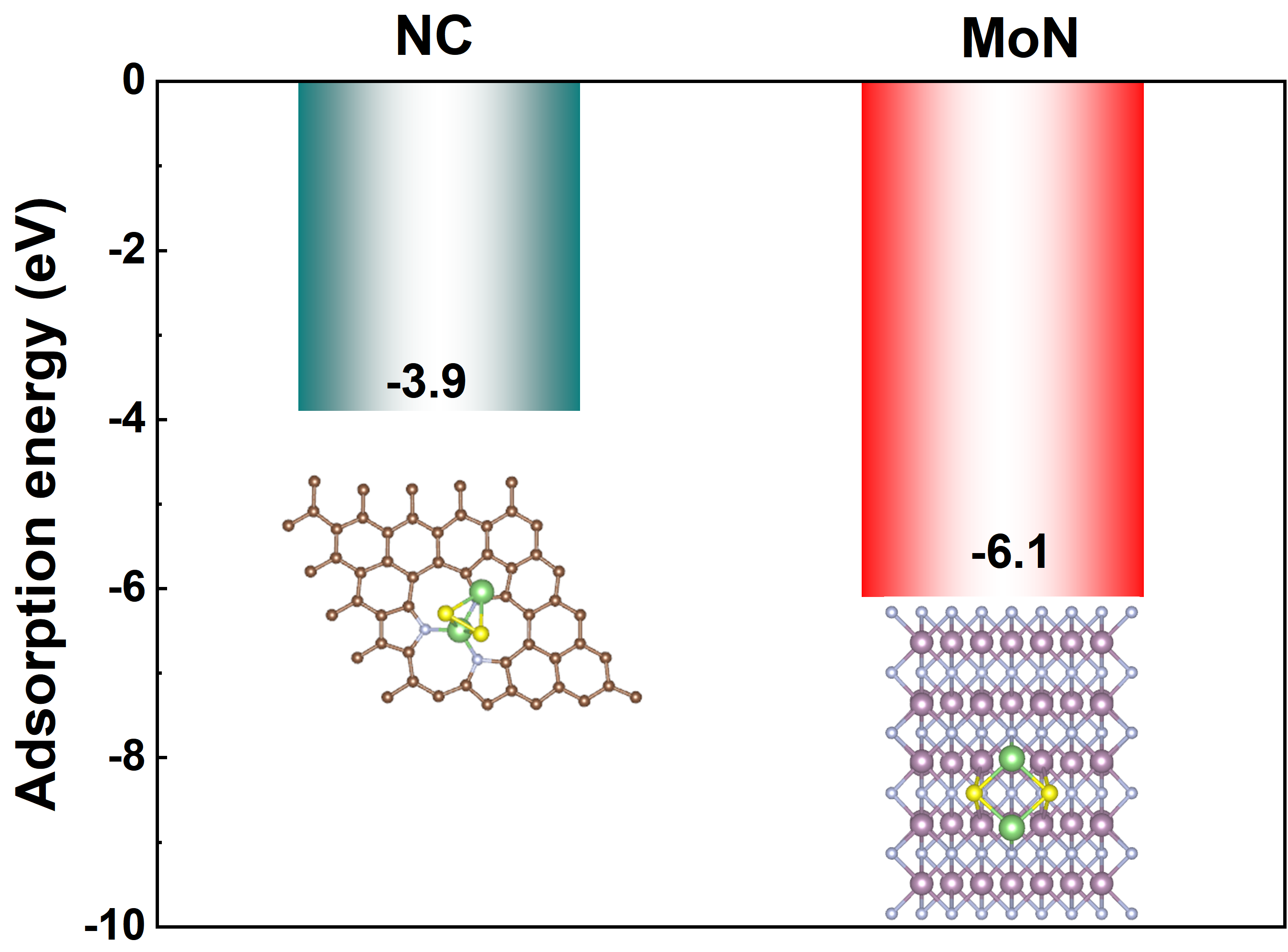


**Figure S9.** Adsorption energies of Li_2_S_2_ on different materials.


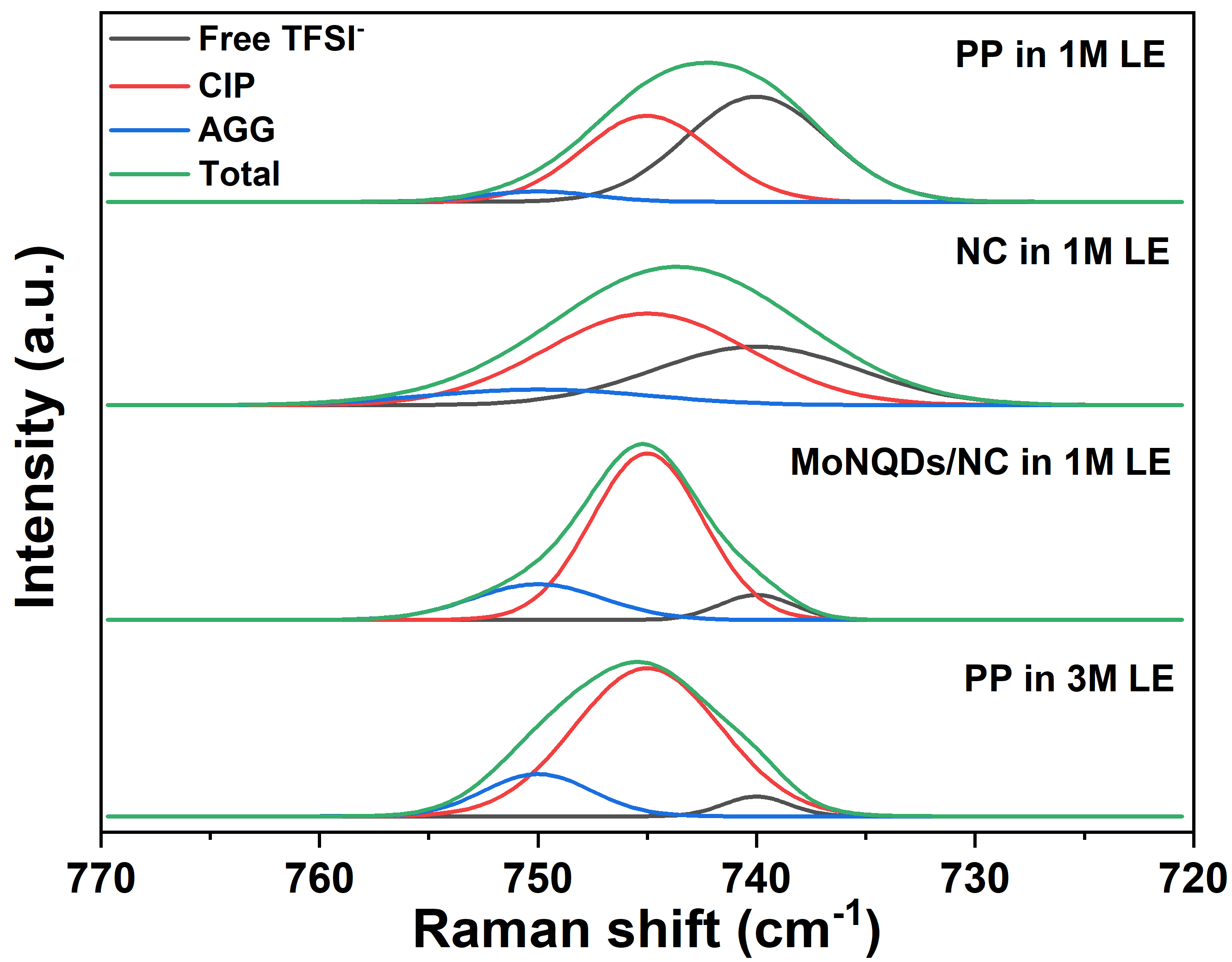


**Figure S10.** Raman spectra and fitting results of PP in 1M LE, NC in 3M LE, MoNQDs/NC in 1M LE and PP in 3M LE.


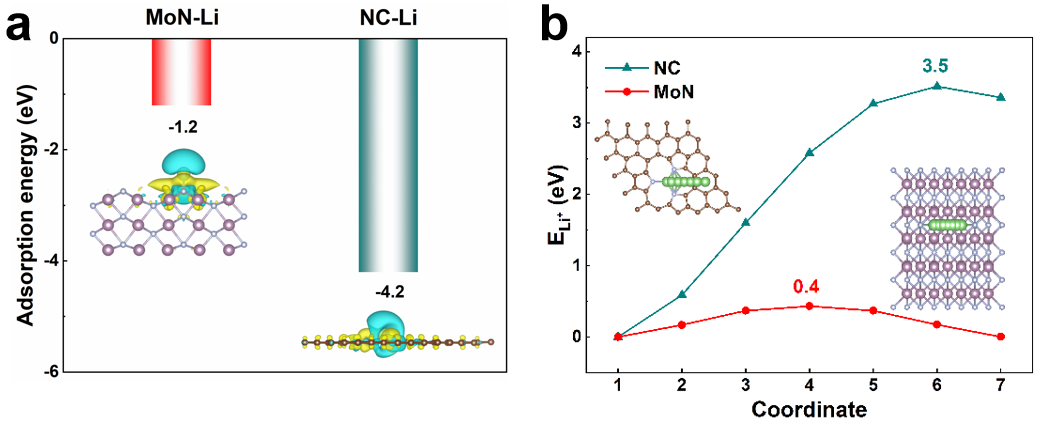


**Figure S11.** a) Adsorption energy and adsorption modeling of Li atom on materials. b) Energy profiles for Li diffusion.


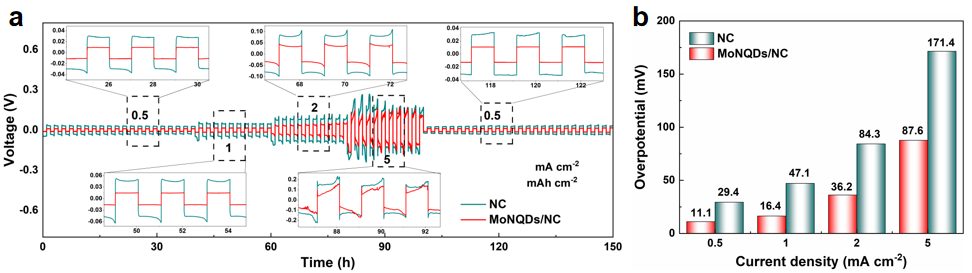


**Figure S12.** a) Rate performance and b) the corresponding overpotential of Li||Li symmetric cells.


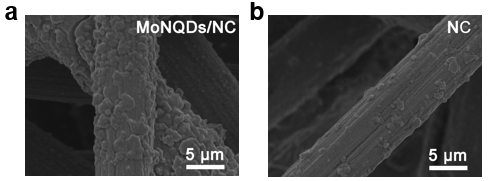


**Figure S13.** SEM images of Li_2_S precipitated on CP with a) MoNQDs/NC and b) NC.


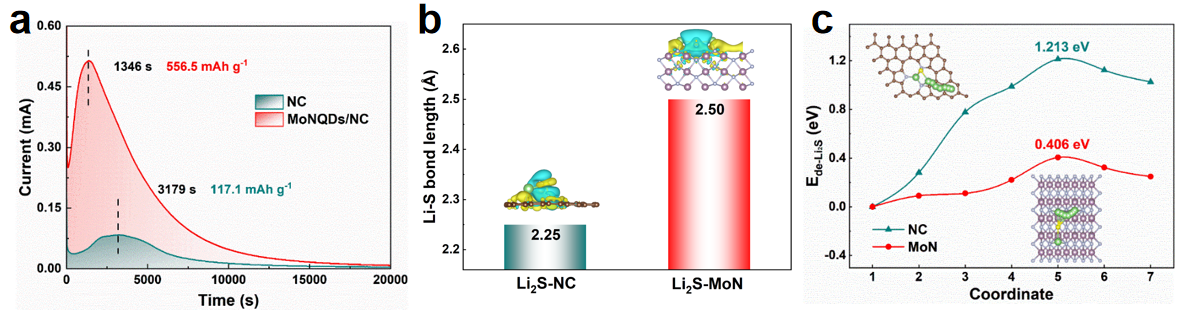


**Figure S14.** a) Dissolution profile of Li_2_S. b) Comparison of Li-S bond lengths in adsorbed Li_2_S. c) Energy profiles of the decomposition of Li_2_S.


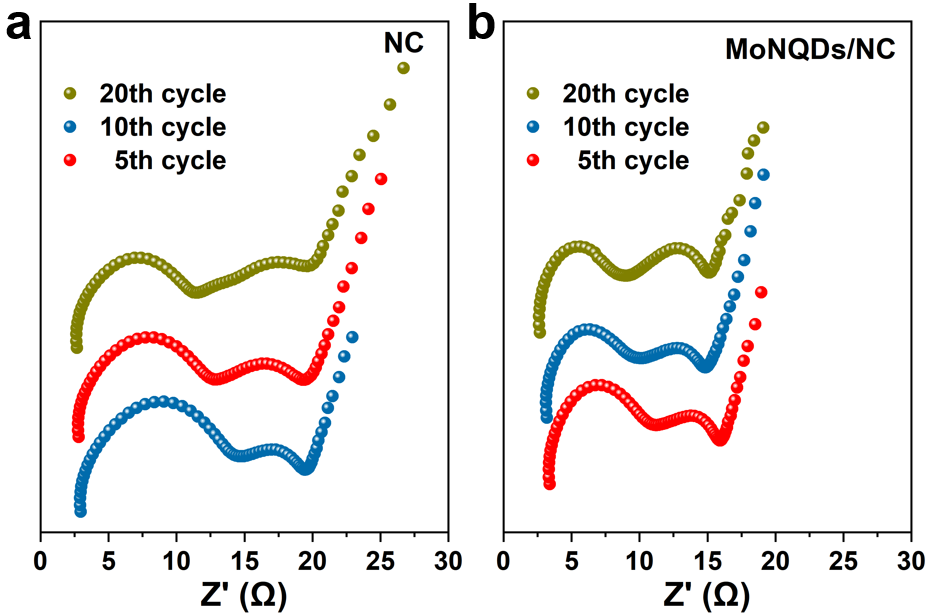


**Figure S15.** Nyquist plots of batteries with a) NC separators and b) MoNQDs/NC separators at different cycle numbers.

**
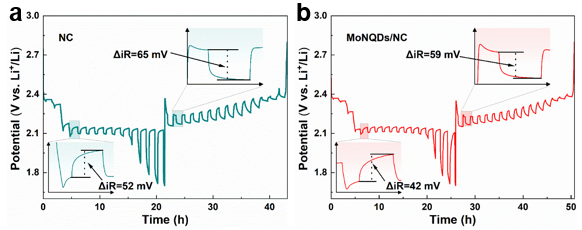
**

**Figure S16.** GITT curves of cells with a) NC and b) MoNQDs/NC.

**
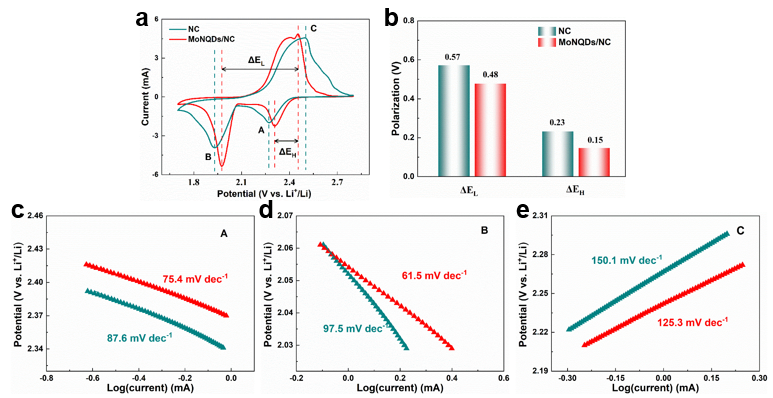
**

**Figure S17.** a) CV curves. b) Corresponding polarization voltage. c-e) Corresponding tafel curves.

**
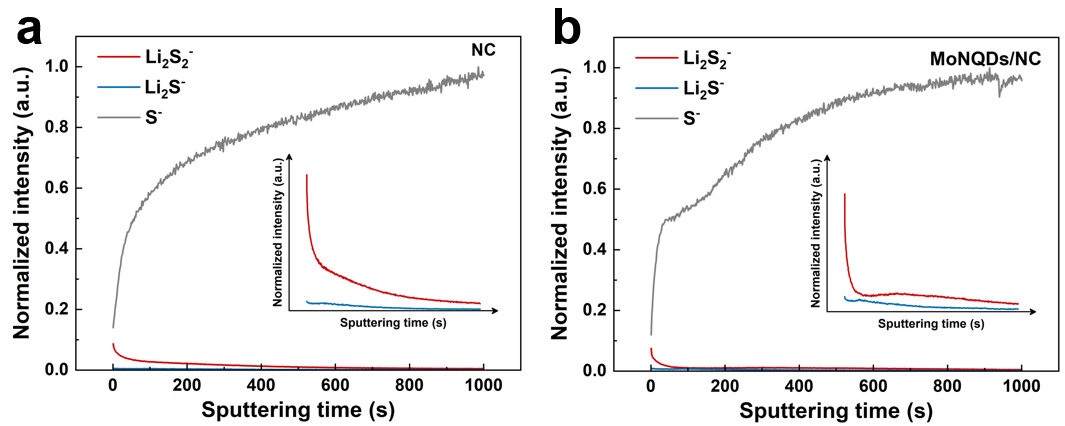
**

**Figure S18.** Depth sputtering profiles of S^−^, Li_2_S^−^ and Li_2_S_2_^−^ of the cathode of the cells with a) NC and b) MoNQDs/NC by ToF-SIMS (the inset is a larger view of the depth sputtering profiles for Li_2_S^−^ and Li_2_S_2_^−^).

**
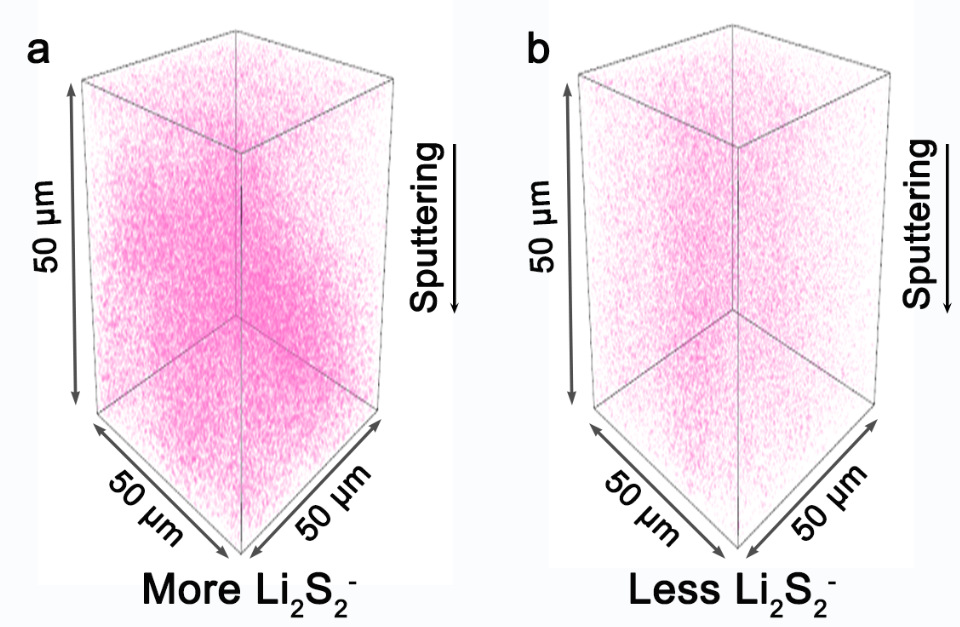
**

**Figure S19.** TOF-SIMS 3D rendering Li_2_S^-^ depth profile images of Li anode of the cells with a) NC and b) MoNQDs/NC.

**
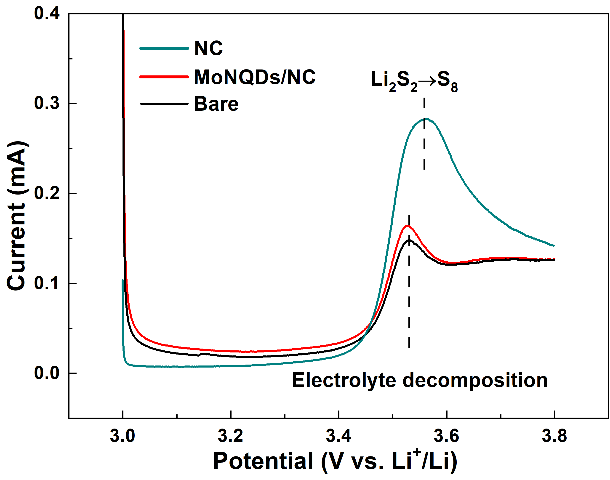
**

**Figure S20.** Response current for voltages ranging from 3 V to 3.8 V.

**

**

**Figure S21.** Cycling performance at 0.2 C

**
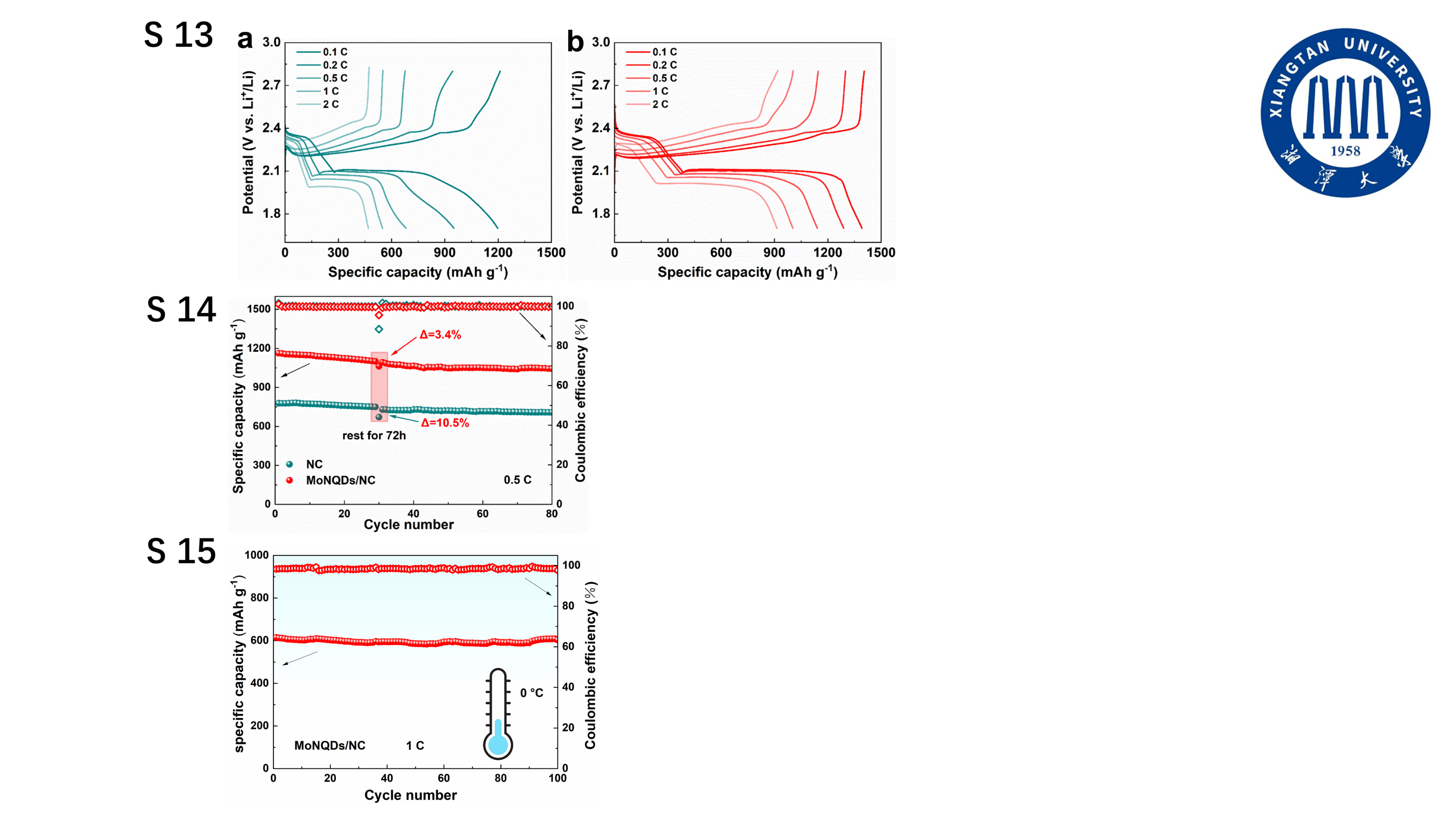
**

**Figure S22.** GCD curves of the rate performance of cells with a) NC and b) MoNQDs/NC.

**

**

**Figure S23.** Self-discharge performance.


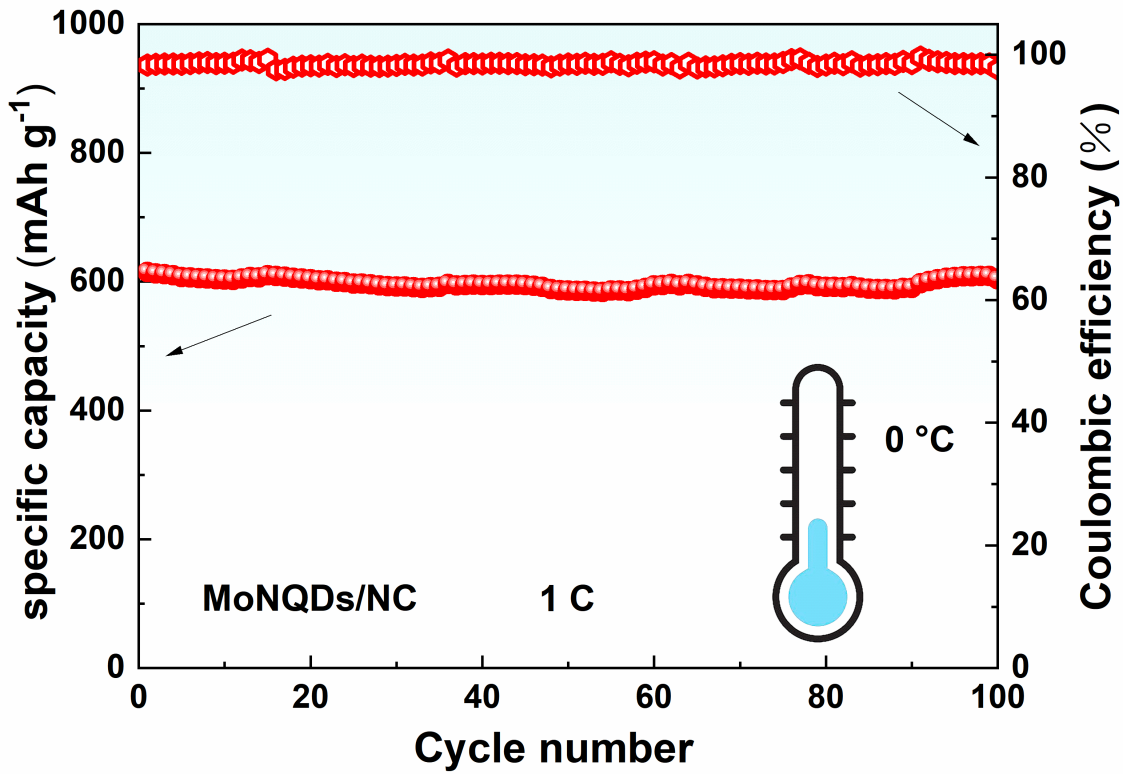


**Figure S24.** Cycling performance at 1 C under 0 ℃.

**Table S1.** Comparison of electrochemical performance with other batteries with high sulfur loading at 0.1 C.

| Materials | Cycle times (cycles) | Sulfur loading (mg cm^-2^) | Areal capacity (mAh cm^-2^) | Capacity decay rate (%) | Ref |
| --- | --- | --- | --- | --- | --- |
| MoNQDs/NC | 50 | 9.1 | 8.3 | 0.012 | This work |
| PSN/MXene | 50 | 5.2 | ~5.2 | ~0.385 | ^[6]^ |
| MoB | 90 | 3.2 | 3.7 | ~0.331 | ^[7]^ |
| Co_5.47_N@NC | 40 | 5.7 | ~6.2 | ~0.427 | ^[8]^ |
| TiNOCF | 120 | 10.0 | 10.2 | 0.149 | ^[9]^ |
| V-MoS_2_@CNT | 65 | 3.8 | ~4.0 | ~0.308 | ^[10]^ |
| Sr-LNO-S | 80 | ~5.4 | ~4.4 | ~0.279 | ^[11]^ |
| TiN-MXene-Co@CNTs | 100 | 5.0 | ~4.9 | ~0.124 | ^[12]^ |
| RuP_2_-RuP@NPSC | 100 | 6.3 | 5.7 | 0.120 | ^[13]^ |
| Co_1_/NCLC | 50 | 7.5 | 7.0 | 0.142 | ^[14]^ |

**References**

[1] a) G. Kresse, J. Furthmüller, *Physical Review B* **1996**, 54, 11169; b) G. Kresse, J. Furthmüller, *Computational Materials Science* **1996**, 6, 15.

[2] J. P. Perdew, K. Burke, M. Ernzerhof, *Physical Review Letters* **1996**, 77, 3865.

[3] P. E. Blöchl, *Physical Review B* **1994**, 50, 17953.

[4] S. Grimme, J. Antony, S. Ehrlich, H. Krieg, *The Journal of Chemical Physics* **2010**, 132.

[5] G. Henkelman, B. P. Uberuaga, H. Jónsson, *The Journal of Chemical Physics* **2000**, 113, 9901.

[6] Z.-H. Luo, M. Zheng, M.-X. Zhou, X.-T. Sheng, X.-L. Chen, J.-J. Shao, T.-S. Wang, G. Zhou, *Advanced Materials* **2025**, 2417321.

[7] J. Pu, S. Fan, Z. Shen, J. Yin, Y. Tan, K. Zhang, B. Wu, G. Hong, Y. Yao, *Advanced Functional Materials* **2025**, 2424215.

[8] M. Li, H. Liu, Z. Cheng, J. He, H. Li, L. Zhang, T. Liu, X. Wang, P. Wang, Z. Liu, G. Cui, *Advanced Energy Materials* **2025**, 2405766.

[9] L. Ji, D. Yang, J. Xue, M. Jia, T. Wu, Q. Zhuang, Y. Zhang, J. Liu, Y. Zhang, *Advanced Energy Materials* **2025**, 2404738.

[10] K. Wang, Y. Wang, J. Wang, H. Wang, C. Ding, Z. Zheng, Y. Liu, Z. Luo, Y. Ding, *Advanced Functional Materials* **2025**, 2422689.

[11] R. Qi, L. Zhao, P. Liu, Y. Zhen, X. Fu, X. Li, Y. Cui, T. Cai, Z. Yan, Q. Xue, W. Xing, *Energy Storage Materials* **2025**, 75, 104065.

[12] X. Zuo, L. Wang, M. Zhen, T. You, D. Liu, Y. Zhang, *Angewandte Chemie International Edition* **2024**, 63, e202408026.

[13] H. Song, T. T. Nguyen, R. Chu, Y. Bai, N. H. Kim, J. H. Lee, *Nano Energy* **2024**, 128, 109859.

[14] F. Cao, X. Zhang, Z. Jin, J. Zhang, Z. Tian, D. Kong, Y. Li, Y. Li, L. Zhi, *Advanced Energy Materials* **2024**, 14, 2303893.
